# Supplementary material for: Chemotaxis to plant defense compounds in phytopathogens
Source: PLoS Pathog. 2026 May 20;22(5):e1014240. doi: 10.1371/journal.ppat.1014240 (PMC13215616; doi:10.1371/journal.ppat.1014240)
Supplement: S1 Fig — A) Schematic view of the locus encoding the four chemoreceptor genes under investigation. The red brackets indicate the position of primer pairs for the analyses shown below. B) For each region, three PCR analyses were carried out: C: positive control with genomic DNA as template; -: negative control with no reverse transcriptase; + : RT-PCR on cDNA. Oligonucleotides amplifying the constitutive gene gyrB were used as a positive control for the reverse transcription-polymerase chain reaction. Samples for RNA isolation were taken at mid-logarithmic phase like the cells used for the chemotaxis assays. (DOCX) [file ppat.1014240.s001.docx]

**A**

**B**

**S1 Fig.** **Transcript analysis by RT-PCR using primers designed to span the intergenic region between two adjacent genes.** **A**) Schematic view of the locus encoding the four chemoreceptor genes under investigation. The red brackets indicate the position of primer pairs for the analyses shown below. **B**) For each region, three PCR analyses were carried out: C: positive control with genomic DNA as template; -: negative control with no reverse transcriptase; +: RT-PCR on cDNA. Oligonucleotides amplifying the constitutive gene *gyrB* were used as a positive control for the reverse transcription-polymerase chain reaction. Samples for RNA isolation were taken at mid-logarithmic phase like the cells used for the chemotaxis assays.
