# Supplementary material for: Chemotaxis to plant defense compounds in phytopathogens
Source: PLoS Pathog. 2026 May 20;22(5):e1014240. doi: 10.1371/journal.ppat.1014240 (PMC13215616; doi:10.1371/journal.ppat.1014240)
Supplement: S2 Fig — PCR analyses were performed using internal primers pairs for each gene: C, positive control with genomic DNA as template; + , RT-PCR on cDNA; -, negative control with no reverse transcriptase. The constitutively expressed gene gyrB was used as a positive control. The following primer pairs were used: RS21440-FW-RT-PCR/RS21440-RV-RT-PCR, RS21445-FW-RT-PCR/RS21445-RV-RT-PCR, RS21455-NdeI-F/RS21455-PstI-R and ECA-gyrB-F-qPCR/ECA-gyrB-R-qPCR (S8 Table). Samples for RNA isolation were taken at mid-logarithmic phase, the same experimental conditions used for the chemotaxis assays, and correspond to the same samples used in Fig 1B. (DOCX) [file ppat.1014240.s002.docx]

**
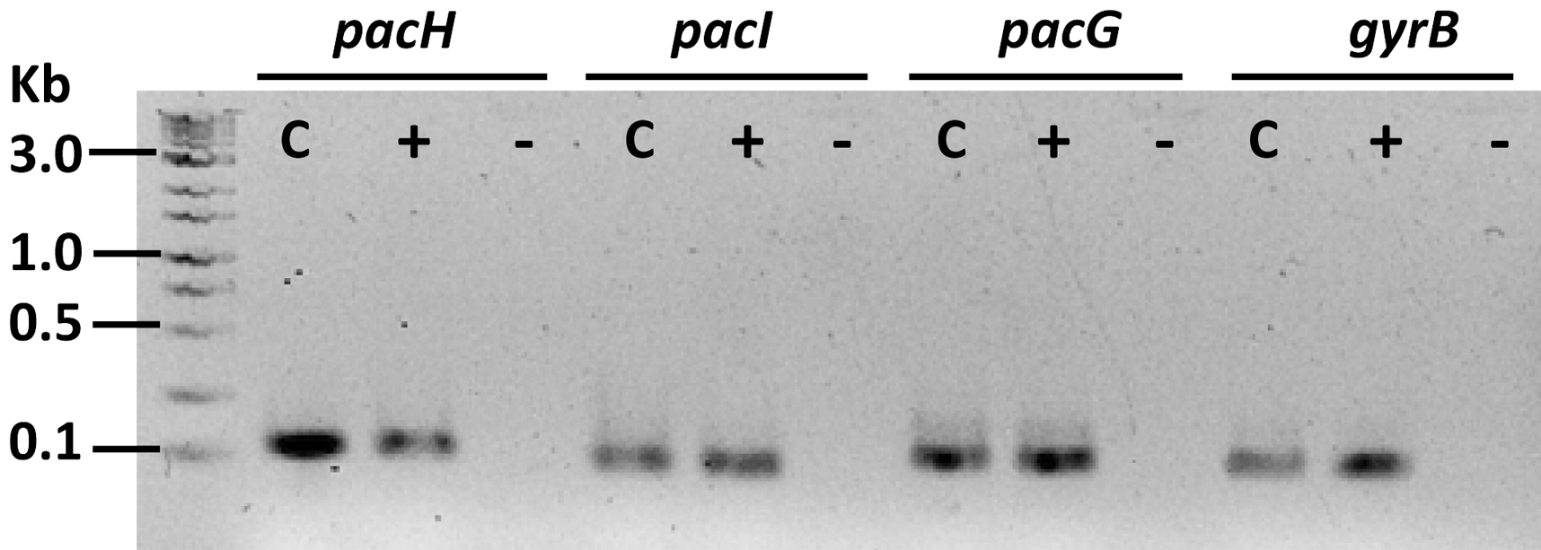
**

**S2 Fig. RT-PCR analysis of** ***pacH, pacI and pacG* gene transcripts in *Pectobacterium atrosepticum* SCRI1043.** PCR analyses were performed using internal primers pairs for each gene: C, positive control with genomic DNA as template; +, RT-PCR on cDNA; -, negative control with no reverse transcriptase. The constitutively expressed gene *gyrB* was used as a positive control. The following primer pairs were used: RS21440-FW-RT-PCR/RS21440-RV-RT-PCR, RS21445-FW-RT-PCR/RS21445-RV-RT-PCR, RS21455-NdeI-F/RS21455-PstI-R and ECA-gyrB-F-qPCR/ECA-gyrB-R-qPCR (Table S8). Samples for RNA isolation were taken at mid-logarithmic phase, the same experimental conditions used for the chemotaxis assays, and correspond to the same samples used in Figure 1B.
