# Supplementary figures and images for: Chemotaxis to plant defense compounds in phytopathogens
Source: PLoS Pathog. 2026 May 20;22(5):e1014240. doi: 10.1371/journal.ppat.1014240 (PMC13215616; doi:10.1371/journal.ppat.1014240)

**S3 Fig. The composition of Biolog compound arrays PM1, PM2A, PM3B, PM4A and PM5.**

**
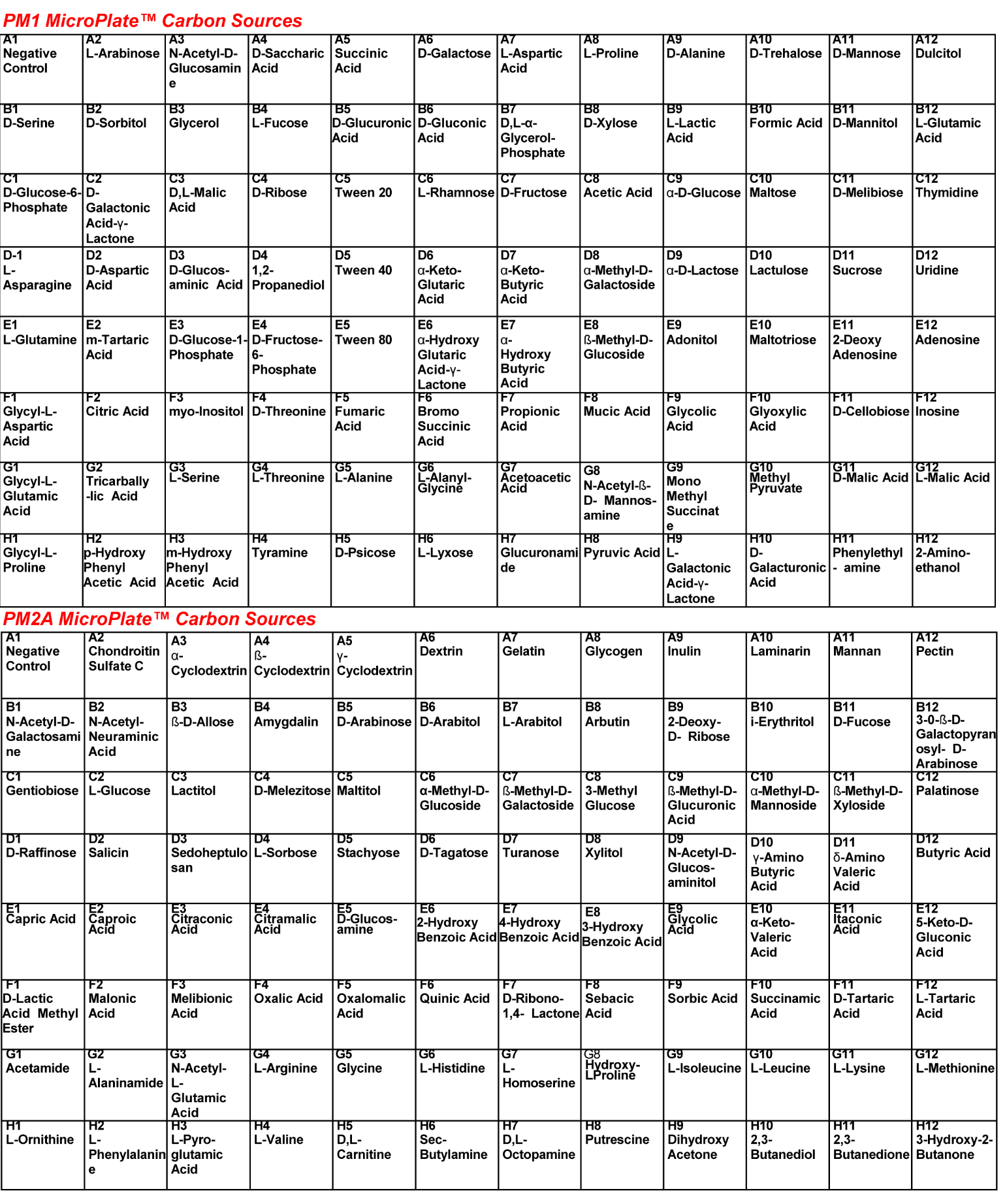
**

**
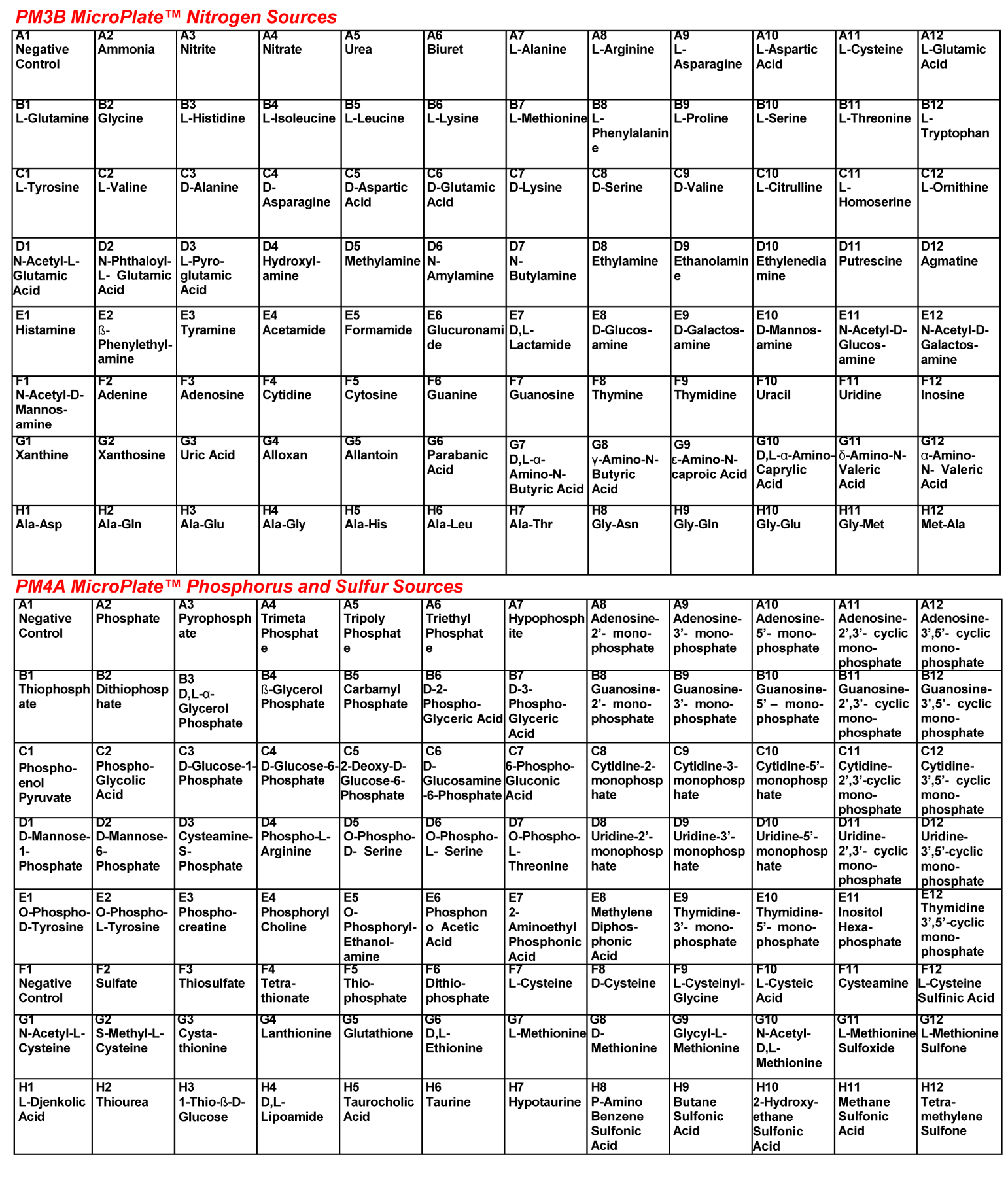
**

Supplement: S3 Fig — (DOCX) [file ppat.1014240.s003.docx]
