# Supplementary material for: Chemotaxis to plant defense compounds in phytopathogens
Source: PLoS Pathog. 2026 May 20;22(5):e1014240. doi: 10.1371/journal.ppat.1014240 (PMC13215616; doi:10.1371/journal.ppat.1014240)
Supplement: S4 Fig — A) Titration of 44 μM PacH-LBD with 12.8 μL aliquots of 5 mM salicylate. B) Titration of 63 μM protein with 12.8 μL aliquots of 5 mM vanillin. Upper panels: Raw titration data. Lower panels: Concentration-normalized and dilution heat-corrected integrated raw data. The derived dissociation constants are provided in Table 1. (DOCX) [file ppat.1014240.s004.docx]

**S4 Fig. Microcalorimetric titration of ECA_RS21440-LBD (PacH-LBD) with salicylate and vanillin.** **A**) Titration of 44 μM PacH-LBD with 12.8 μL aliquots of 5 mM salicylate. **B**) Titration of 63 μM protein with 12.8 μL aliquots of 5 mM vanillin. Upper panels: Raw titration data. Lower panels: Concentration-normalized and dilution heat-corrected integrated raw data. The derived dissociation constants are provided in Table 1.

**

**
