# Supplementary material for: Chemotaxis to plant defense compounds in phytopathogens
Source: PLoS Pathog. 2026 May 20;22(5):e1014240. doi: 10.1371/journal.ppat.1014240 (PMC13215616; doi:10.1371/journal.ppat.1014240)
Supplement: S5 Fig — Shown are the raw data for the titration of 50 µM PacI-LBD with 12.8 µl aliquots of 1 mM capric acid. Heat changes are small and uniform and comparable to the titration of buffer with capric acid, indicative of an absence of binding. The scale on the y-axis corresponds to that of Fig 2B. (DOCX) [file ppat.1014240.s005.docx]

**S5 Fig. Microcalorimetric binding studies of capric acid to PacI-LBD.** Shown are the raw data for the titration of 50 µM PacI-LBD with 12.8 µl aliquots of 1 mM capric acid. Heat changes are small and uniform and comparable to the titration of buffer with capric acid, indicative of an absence of binding. The scale on the y-axis corresponds to that of Fig. 2B.

**
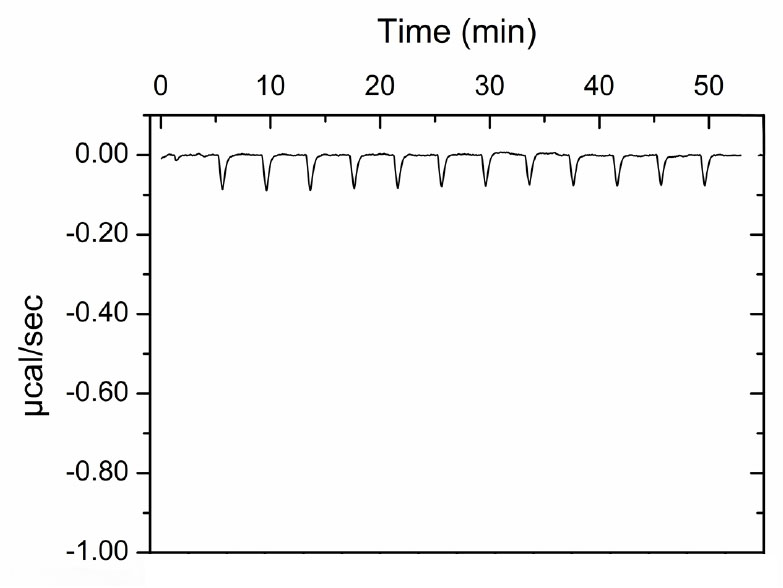
**
