# Supplementary material for: Chemotaxis to plant defense compounds in phytopathogens
Source: PLoS Pathog. 2026 May 20;22(5):e1014240. doi: 10.1371/journal.ppat.1014240 (PMC13215616; doi:10.1371/journal.ppat.1014240)
Supplement: S6 Fig — Proton signal assignments are indicated in the spectra and correspond to the numbering shown in the chemical structures. (DOCX) [file ppat.1014240.s006.docx]

**S6 Fig.** **^1^H-NMR spectra of *p*-coumaroylagmatine and feruloylagmatine (400 MHz, CD_3_OD).** Proton signal assignments are indicated in the spectra and correspond to the numbering shown in the chemical structures.

**
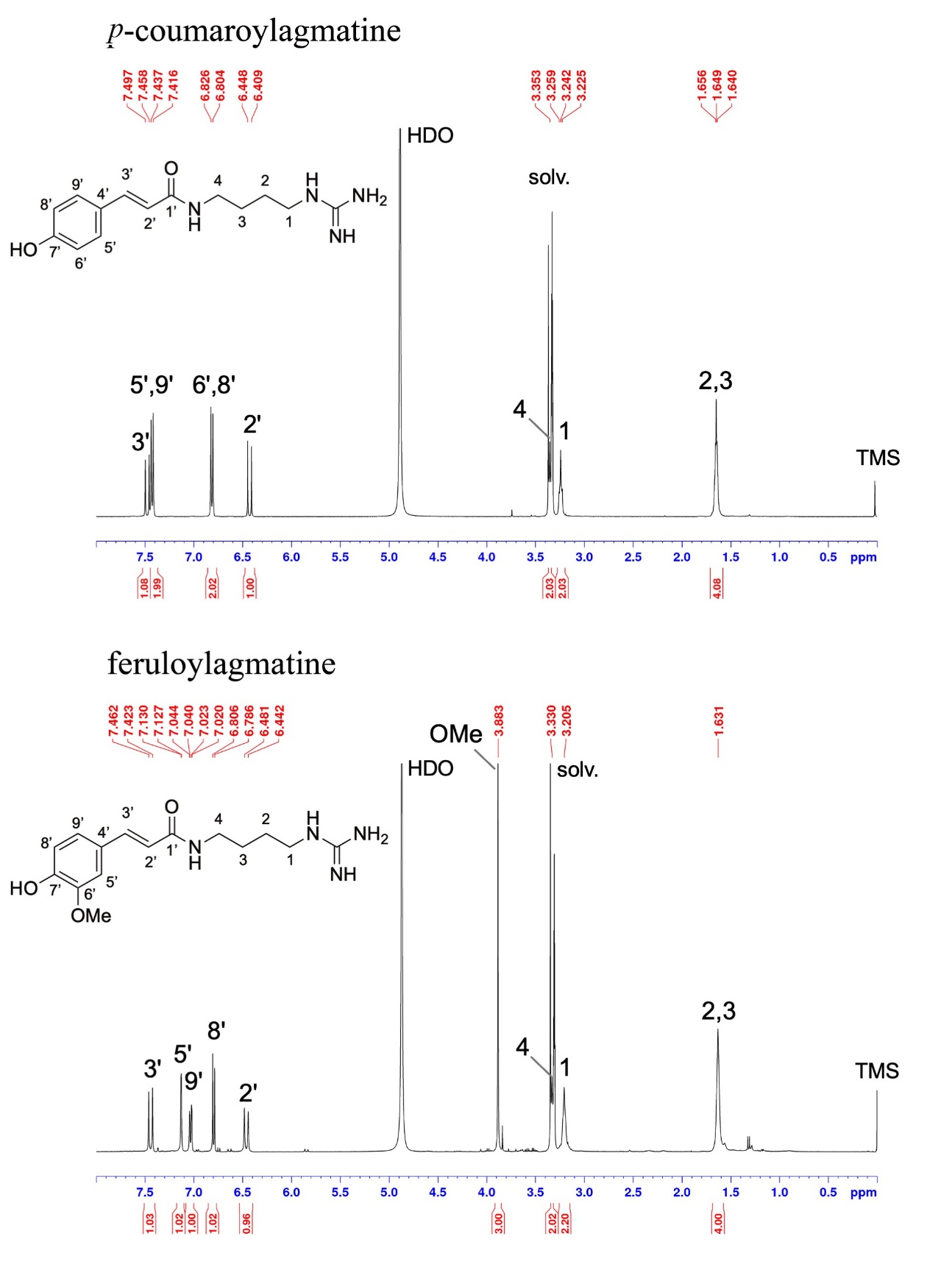
**
