# Supplementary material for: Chemotaxis to plant defense compounds in phytopathogens
Source: PLoS Pathog. 2026 May 20;22(5):e1014240. doi: 10.1371/journal.ppat.1014240 (PMC13215616; doi:10.1371/journal.ppat.1014240)

**S7 Fig. The chemotaxis to vanillin (A), salicylate (B) and benzoate (C) is induced by benzoate.** Quantitative chemotaxis capillary assays of *P. atrosepticum* SCRI1043 to different concentrations of vanillin, salicylate and benzoate when grown in minimal medium (MM) or MM supplemented with 500 µM of vanillin or benzoate. Data have been corrected with the number of bacteria that swam into buffer containing capillaries namely 1,537 (vanillin in MM), 50 (vanillin in MM + vanillin), 2,771 (vanillin in MM + benzoate), 1,537 (salicylate in MM), 2,324 (salicylate in MM + benzoate), 720 (benzoate in MM), and 986 (benzoate in MM + benzoate).

##
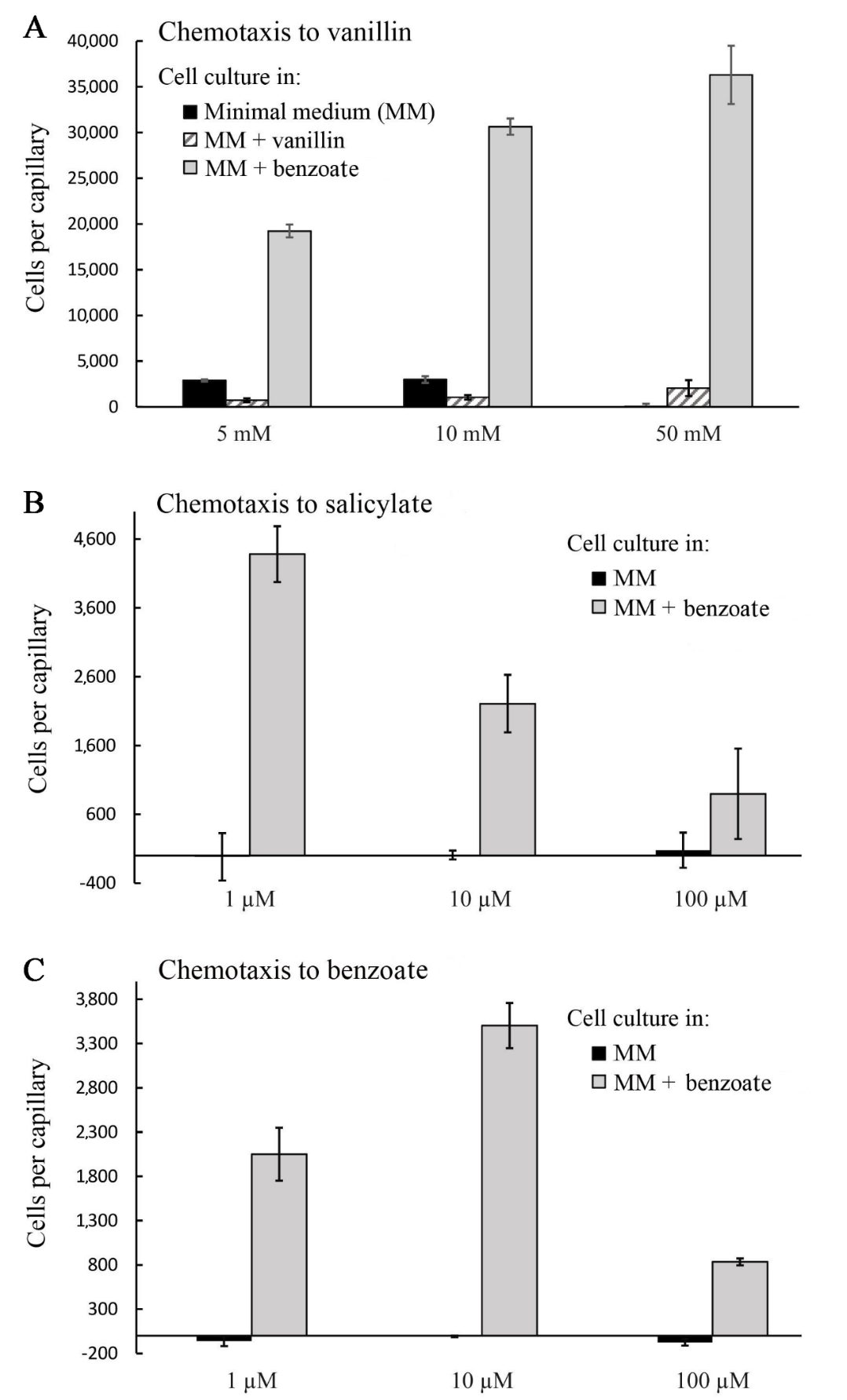

Supplement: S7 Fig — Quantitative chemotaxis capillary assays of P. atrosepticum SCRI1043 to different concentrations of vanillin, salicylate and benzoate when grown in minimal medium (MM) or MM supplemented with 500 µM of vanillin or benzoate. Data have been corrected with the number of bacteria that swam into buffer containing capillaries namely 1,537 (vanillin in MM), 50 (vanillin in MM + vanillin), 2,771 (vanillin in MM + benzoate), 1,537 (salicylate in MM), 2,324 (salicylate in MM + benzoate), 720 (benzoate in MM), and 986 (benzoate in MM + benzoate). (DOCX) [file ppat.1014240.s007.docx]
