# Supplementary material for: Chemotaxis to plant defense compounds in phytopathogens
Source: PLoS Pathog. 2026 May 20;22(5):e1014240. doi: 10.1371/journal.ppat.1014240 (PMC13215616; doi:10.1371/journal.ppat.1014240)
Supplement: S8 Fig — Data have been corrected with the number of bacteria that swam into buffer-containing capillaries, namely 682 (in minimal medium) and 1,197 (in minimal medium + 500 µM benzoate). (DOCX) [file ppat.1014240.s008.docx]

**S8 Fig. Quantitative capillary chemotaxis assays of *P. atrosepticum* SCRI 1043 to 5 mM L-Asp for cells grown in minimal medium and minimal medium containing 500 µM benzoate.** Data have been corrected with the number of bacteria that swam into buffer-containing capillaries, namely 682 (in minimal medium) and 1,197 (in minimal medium + 500 µM benzoate).

**
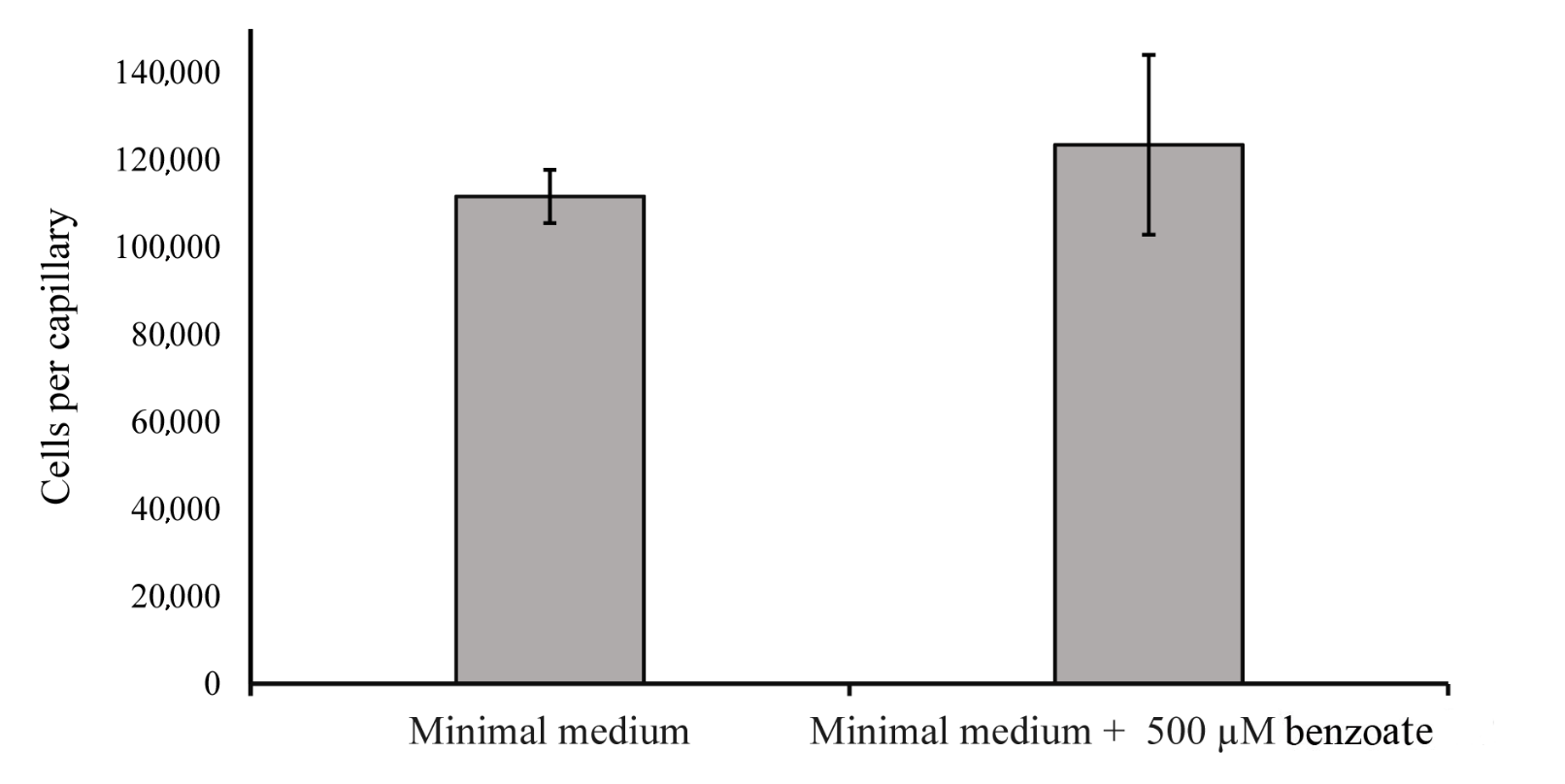
**
