# Supplementary material for: Chemotaxis to plant defense compounds in phytopathogens
Source: PLoS Pathog. 2026 May 20;22(5):e1014240. doi: 10.1371/journal.ppat.1014240 (PMC13215616; doi:10.1371/journal.ppat.1014240)
Supplement: S9 Fig — Strains for experiments shown in panel A were grown in minimal medium and 500 µM benzoate was added 40 minutes after inoculation. Strains for experiments shown in panel B were grown in minimal medium. Data have been corrected with the number of bacteria that swam into buffer containing capillaries, namely 1,197 (wild type, panel A), 1,777 (ΔpacH), 1,675 (ΔpacI), 963 (wild type, panel B) and 918 (ΔpacG). (DOCX) [file ppat.1014240.s009.docx]

**S9 Fig. Quantitative chemotaxis capillary assays of *P. atrosepticum* SCRI1043 strains to 5 mM L-Asp (A) and 0.1 % (w/v) casamino acids (B).** Strains for experiments shown in panel A were grown in minimal medium and 500 µM benzoate was added 40 minutes after inoculation. Strains for experiments shown in panel B were grown in minimal medium. Data have been corrected with the number of bacteria that swam into buffer containing capillaries, namely 1,197 (wild type, panel A), 1,777 (Δ*pacH*), 1,675 (Δ*pacI*), 963 (wild type, panel B) and 918 (Δ*pacG*).
