# Supplementary material for: Chemotaxis to plant defense compounds in phytopathogens
Source: PLoS Pathog. 2026 May 20;22(5):e1014240. doi: 10.1371/journal.ppat.1014240 (PMC13215616; doi:10.1371/journal.ppat.1014240)
Supplement: S10 Fig — A) Quantitative capillary assays of P. atrosepticum SCRI1043 grown aerobically in minimal medium with glucose or agmatine as carbon source or in potato broth. The agmatine concentrations are indicated. Data have been corrected for the number of cells that swam into buffer-only capillaries: 1,685 (glucose as carbon source), 1,462 (agmatine as carbon source) and 1,293 (potato broth). B) Quantitative capillary assays of P. atrosepticum SCRI1043 grown anaerobically in minimal medium with glucose as carbon source. The agmatine concentrations are indicated. Data have been corrected for the number of cells that swam into buffer-only capillaries (690). The means and standard deviations from three biological replicates conducted in triplicate are shown. (DOCX) [file ppat.1014240.s010.docx]

**S10 Fig. Chemotaxis to agmatine in under aerobic (A) and anaerobic (B) growth conditions**. **A**) Quantitative capillary assays of *P. atrosepticum* SCRI1043 grown aerobically in minimal medium with glucose or agmatine as carbon source or in potato broth. The agmatine concentrations are indicated. Data have been corrected for the number of cells that swam into buffer-only capillaries: 1,685 (glucose as carbon source), 1,462 (agmatine as carbon source) and 1,293 (potato broth). **B**) Quantitative capillary assays of *P. atrosepticum* SCRI1043 grown anaerobically in minimal medium with glucose as carbon source. The agmatine concentrations are indicated. Data have been corrected for the number of cells that swam into buffer-only capillaries (690). The means and standard deviations from three biological replicates conducted in triplicate are shown.

**
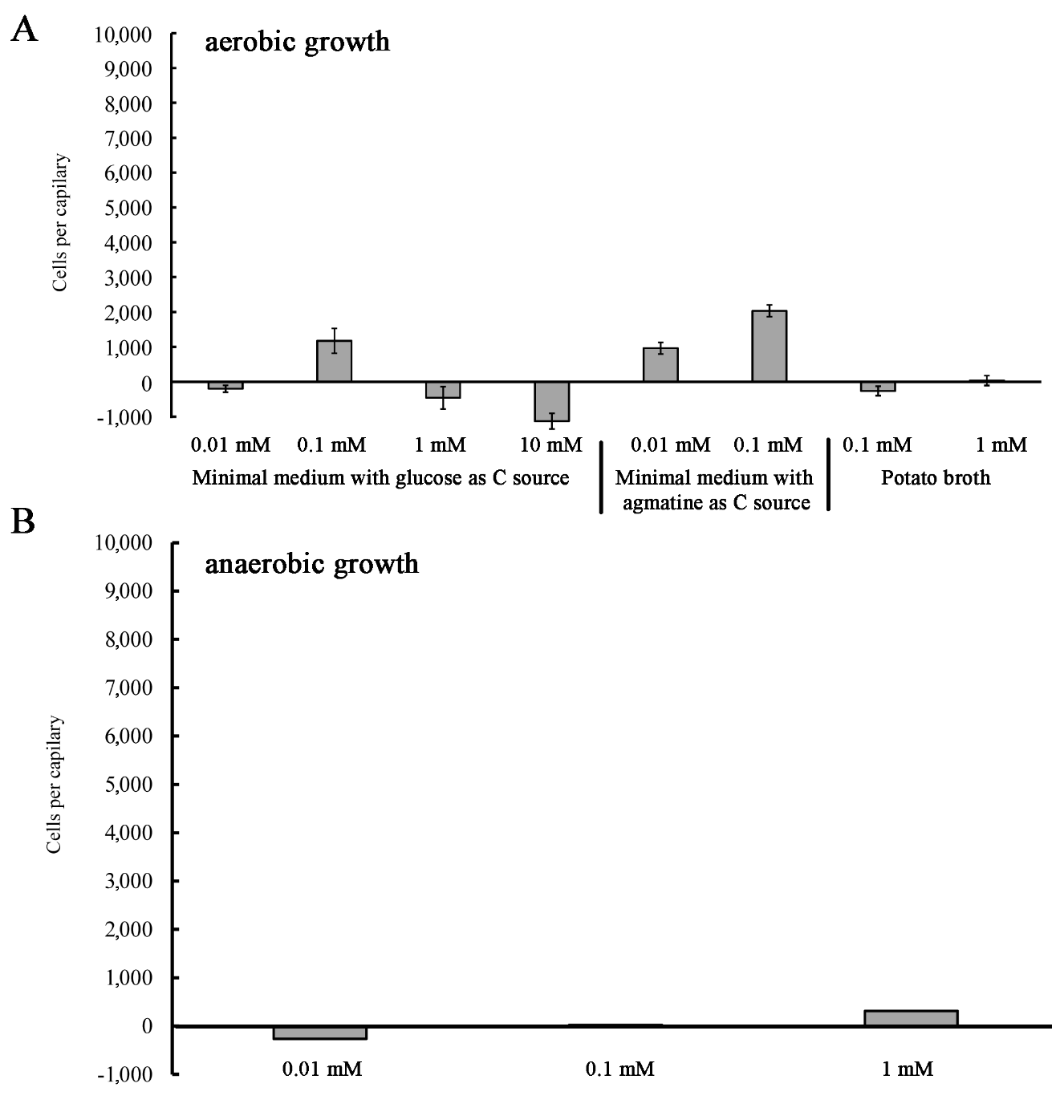
**
