# Supplementary material for: Chemotaxis to plant defense compounds in phytopathogens
Source: PLoS Pathog. 2026 May 20;22(5):e1014240. doi: 10.1371/journal.ppat.1014240 (PMC13215616; doi:10.1371/journal.ppat.1014240)
Supplement: S11 Fig — Tuber-slice assays of P. atrosepticum SCRI1043 strains. Wild type (WT) or pacG and pacHIG mutants harbouring either the empty expression plasmid pBBR empty or the pBBRpacG expression plasmid were used to infect potato tubers. Inoculation with a MgSO4 solution served as control. Representative images of the phenotypes observed are shown in the upper part. The statistical analysis of one of three experiments is shown in the lower part. *** p < 0.0005 in unpaired t-test. (DOCX) [file ppat.1014240.s011.docx]

**S11 Fig. Complementation experiments of plant infection assays.** Tuber-slice assays of *P. atrosepticum* SCRI1043 strains. Wild type (WT) or *pacG* and *pacHIG* mutants harbouring either the empty expression plasmid pBBR empty or the pacBBR*pacG* expression plasmid were used to infect potato tubers. Inoculation with a MgSO_4_ solution served as control. Representative images of the phenotypes observed are shown in the upper part. The statistical analysis of one of three experiments is shown in the lower part. *** p < 0.0005 in unpaired T-test.

**
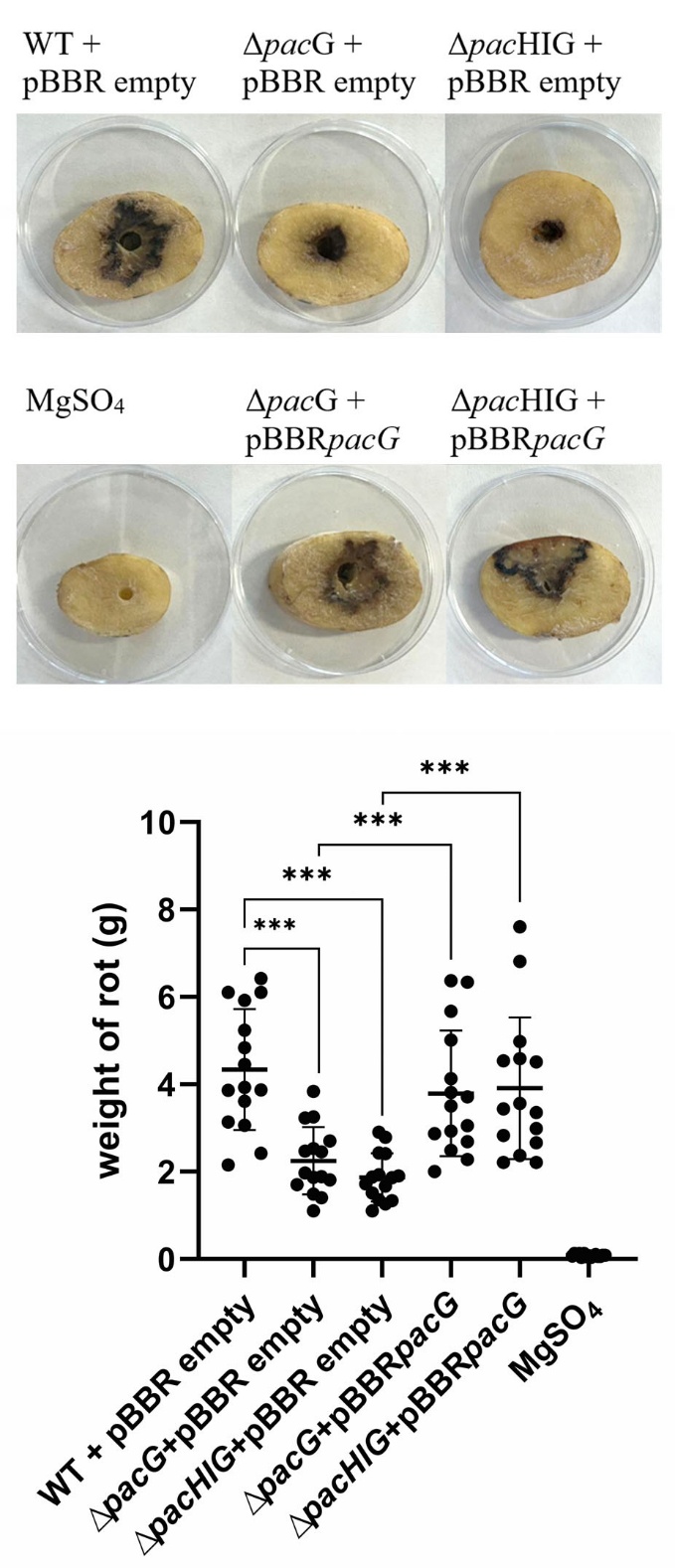
**
