# Supplementary material for: Chemotaxis to plant defense compounds in phytopathogens
Source: PLoS Pathog. 2026 May 20;22(5):e1014240. doi: 10.1371/journal.ppat.1014240 (PMC13215616; doi:10.1371/journal.ppat.1014240)

**S12 Fig. Soft root tissue production in potato inoculated with different *P. atrosepticum* SCRI1043 strains.** Results from tuber slice assays. Chemoreceptors PacF, PacN and PacP were shown to mediate chemotaxis to formate (1), nitrate (2) and phosphorylated compounds (3), respectively. MgSO_4_ was added as a negative control. The p-value was determined by the unpaired T-test: **** p < 0.0001; ns: not significant.


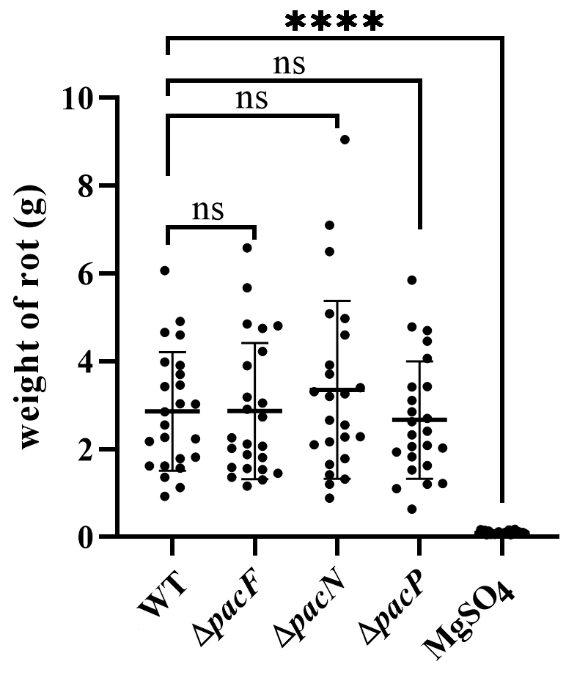

Supplement: S12 Fig — Results from tuber slice assays. Chemoreceptors PacF, PacN and PacP were shown to mediate chemotaxis to formate (1), nitrate (2) and phosphorylated compounds (3), respectively. MgSO4 was added as a negative control. The p-value was determined by the unpaired t-test: **** p < 0.0001; ns: not significant. (DOCX) [file ppat.1014240.s012.docx]
