# Supplementary material for: Chemotaxis to plant defense compounds in phytopathogens
Source: PLoS Pathog. 2026 May 20;22(5):e1014240. doi: 10.1371/journal.ppat.1014240 (PMC13215616; doi:10.1371/journal.ppat.1014240)
Supplement: S13 Fig — Data are means and standard deviations from three biological replicates. (DOCX) [file ppat.1014240.s013.docx]

**S13 Fig. Growth experiments of *P. atrosepticum* SCRI1043, a mutant deficient in the *pacG* gene, and the mutant strain complemented with a plasmid harboring the *pacG* gene in minimal medium supplemented with 0.2 % (w/v) glucose as sole C-source (A) and LB medium (B).** Data are means and standard deviations from three biological replicates.

**
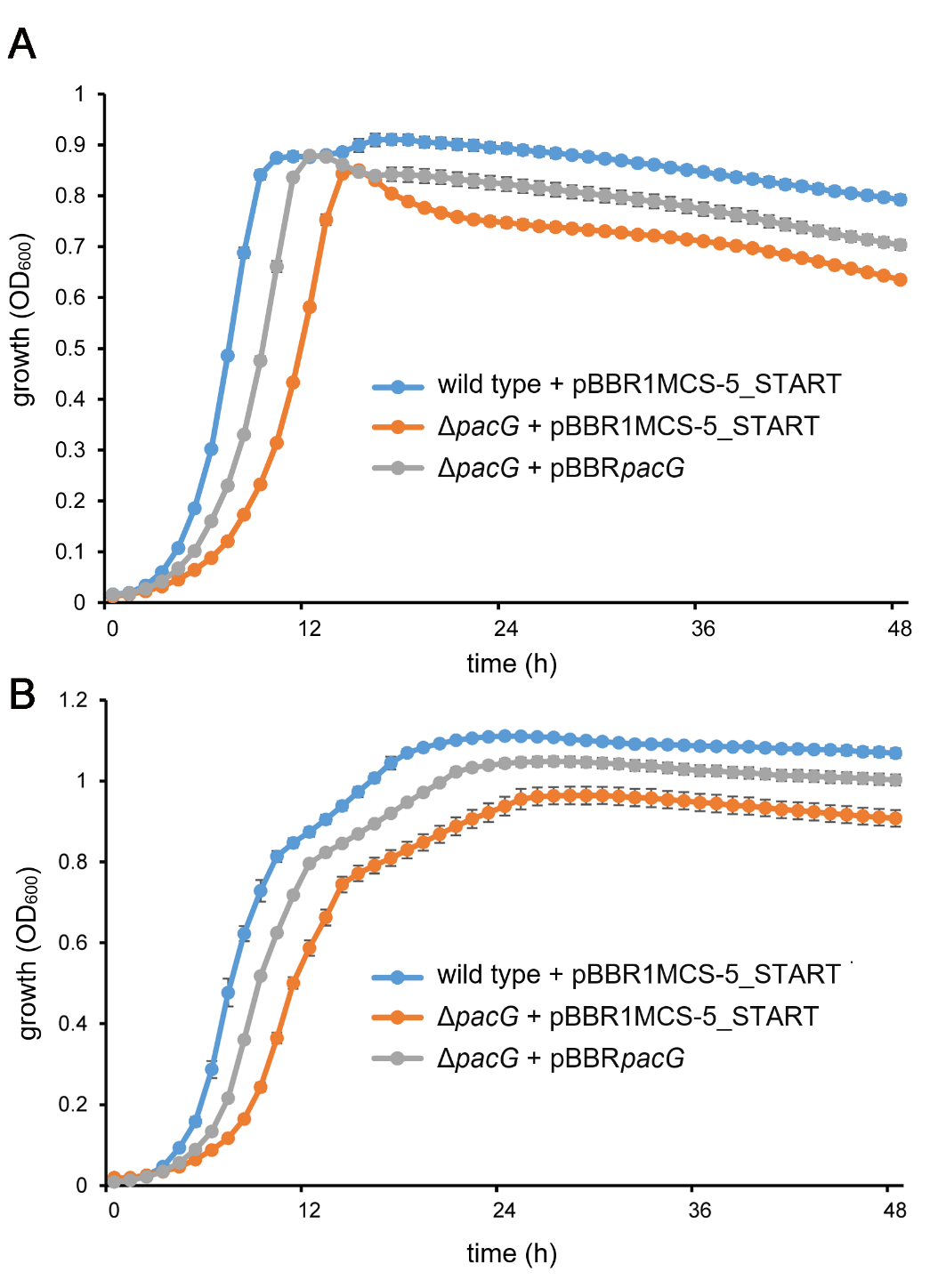
**
