# Supplementary material for: Chemotaxis to plant defense compounds in phytopathogens
Source: PLoS Pathog. 2026 May 20;22(5):e1014240. doi: 10.1371/journal.ppat.1014240 (PMC13215616; doi:10.1371/journal.ppat.1014240)
Supplement: S14 Fig — Experiments were conducted with the wild type (WT) strain, the mutant in the pacG gene (∆pacG) and the mutant complemented with a plasmid harboring the pacG gene (compl). A) Skimmed milk agar plate assay of protease production. B) Pectate lyase assay. C) Cellulase assay. (DOCX) [file ppat.1014240.s014.docx]

**S14 Fig. Assessment of the impact of the deletion of *pacG* on the production of plant cell wall–degrading enzymes.** Experiments were conducted with the wild type (WT) strain, the mutant in the *pacG* gene (∆*pacG*) and the mutant complemented with a plasmid harboring the *pacG* gene (compl). **A)** Skimmed milk agar plate assay of protease production. **B)** Pectate lyase assay. **C)** Cellulase assay.

**
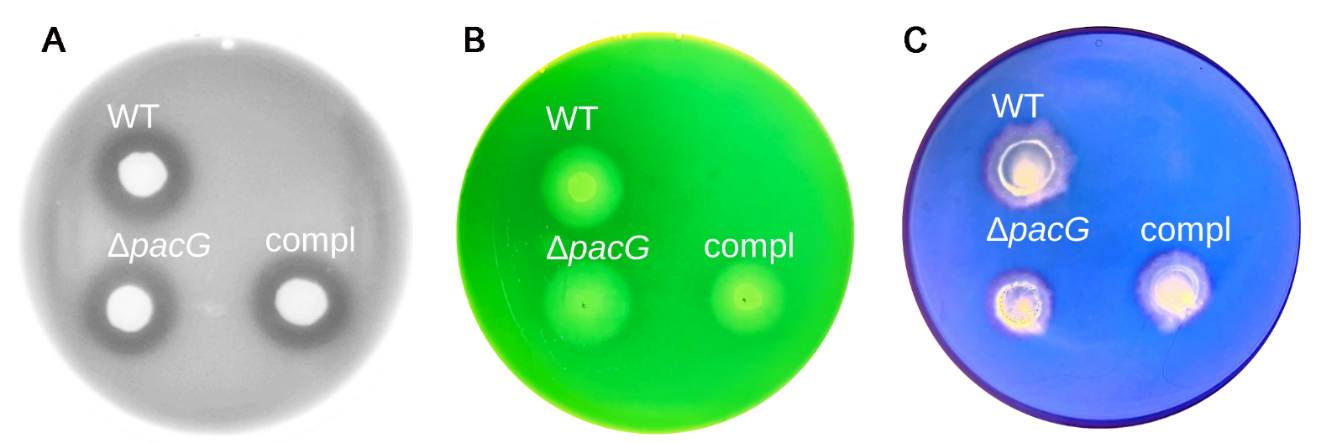
**
