# Supplementary material for: Chemotaxis to plant defense compounds in phytopathogens
Source: PLoS Pathog. 2026 May 20;22(5):e1014240. doi: 10.1371/journal.ppat.1014240 (PMC13215616; doi:10.1371/journal.ppat.1014240)

**S15 Fig. Three-dimensional structure of PacG-LBD.** Although the protein was co-crystallized with agmatine and *p*-coumaroylagmatine, no electron density accounting for these compounds was visualized. Instead, the protein dimer contained four sulfate molecules, of which two in the binding pocket. Sulfate molecules are shown in stick mode. The |2Fo-Fc| map is contoured at 2.0 σ (in blue). The protein was crystallized in the presence of 2 M (NH_4_)_2_SO_4_.


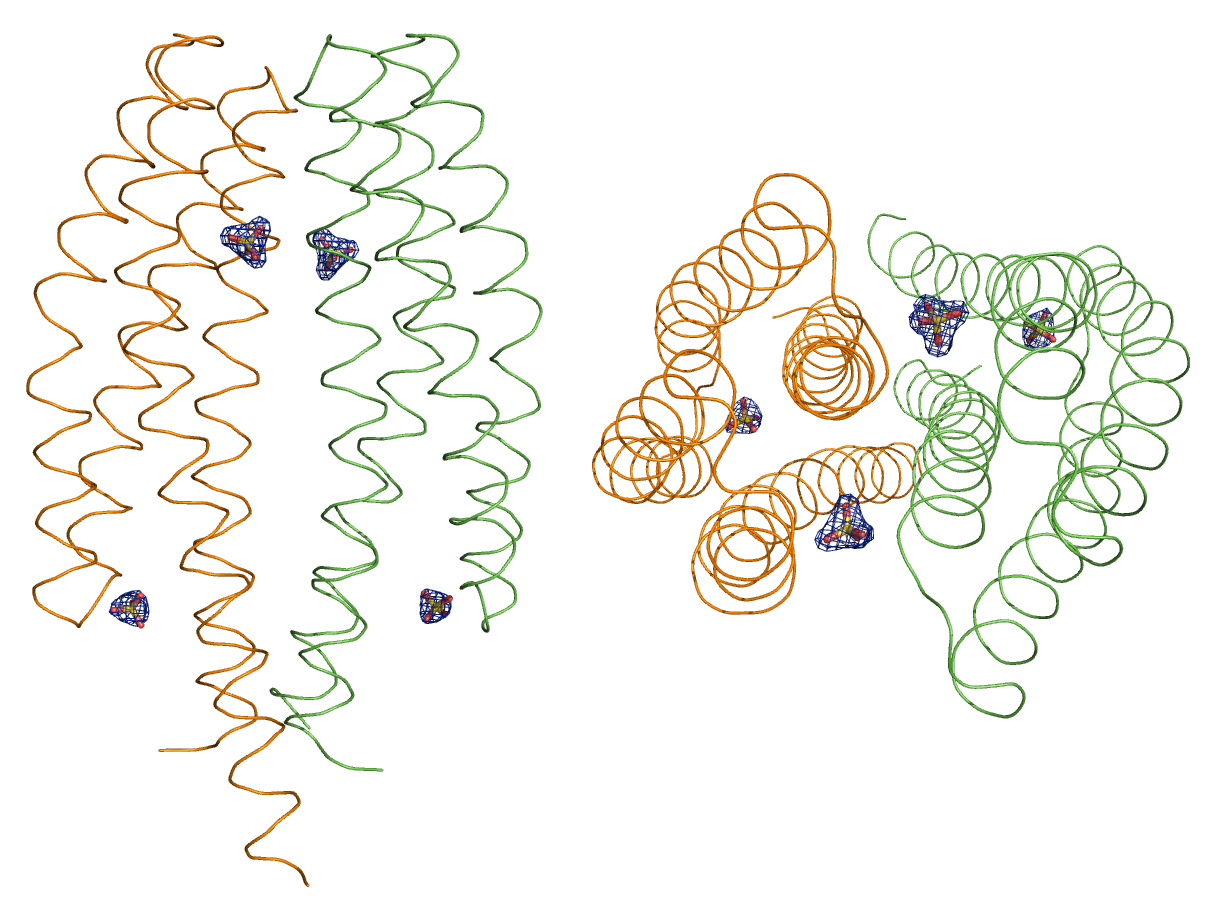

Supplement: S15 Fig — Although the protein was co-crystallized with agmatine and p-coumaroylagmatine, no electron density accounting for these compounds was visualized. Instead, the protein dimer contained four sulfate molecules, of which two in the binding pocket. Sulfate molecules are shown in stick mode. The |2Fo-Fc| map is contoured at 2.0 σ (in blue). The protein was crystallized in the presence of 2 M (NH4)2SO4. (DOCX) [file ppat.1014240.s015.docx]
