# Supplementary material for: Chemotaxis to plant defense compounds in phytopathogens
Source: PLoS Pathog. 2026 May 20;22(5):e1014240. doi: 10.1371/journal.ppat.1014240 (PMC13215616; doi:10.1371/journal.ppat.1014240)
Supplement: S16 Fig — The monomers of the dimer are colored differently. (DOCX) [file ppat.1014240.s016.docx]

**S16 Fig. Molecular docking of feruloylagmatine (left) and *p*-coumaroylagmatine (right) to the structure of PacG-LBD.** The monomers of the dimer are colored differently.

**
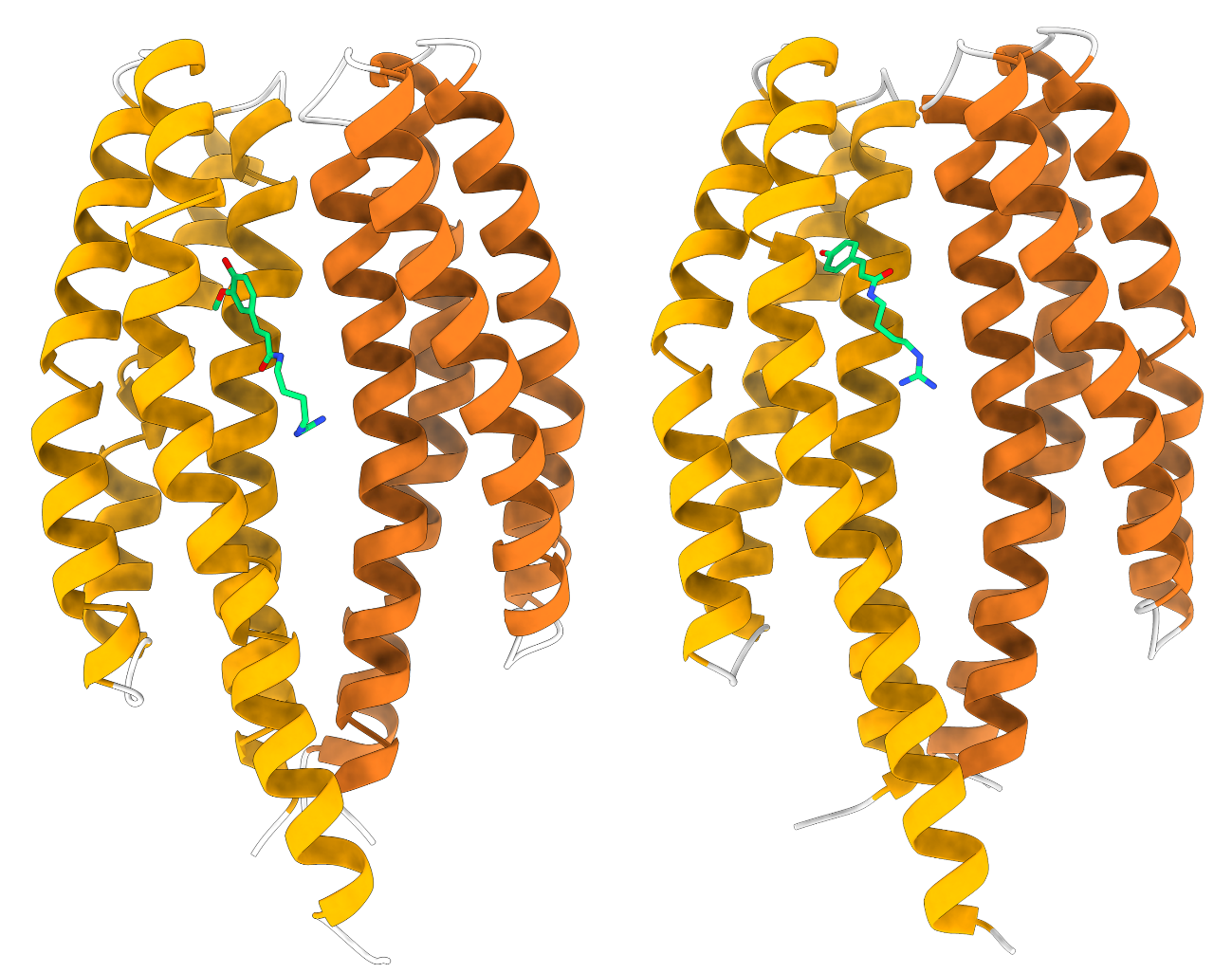
**
