# Supplementary material for: Chemotaxis to plant defense compounds in phytopathogens
Source: PLoS Pathog. 2026 May 20;22(5):e1014240. doi: 10.1371/journal.ppat.1014240 (PMC13215616; doi:10.1371/journal.ppat.1014240)
Supplement: S17 Fig — A protein-protein BLAST (blastp) (4) search in the NCBI non-redundant protein sequence database was conducted using the PacG-LBD sequence as query. Default parameters were used, and sequences of the taxid Pectobacterium (taxid:122277) were excluded. The alignment was done using the CLUSTALW algorithm of the NPS@ software (5). The Gonnet protein weight matrix was used; gap opening and gap extension penalties were 10.0 and 0.1, respectively. Residues in red are identical, green highly similar and blue weakly similar. Five amino acids present in the ligand binding pocket are fully conserved and shaded in yellow. The corresponding amino acids are circled in Fig 7B. (DOCX) [file ppat.1014240.s017.docx]

**S17 Fig. Alignment of the PacG-LBD sequence with those of homologous domains.** A protein-protein BLAST (blastp)(4) search in the NCBI non-redundant protein sequence database was conducted using the PacG-LBD sequence as query. Default parameters were used, and sequences of the taxid *Pectobacterium* (taxid:122277) were excluded. The alignment was done using the CLUSTALW algorithm of the NPS@ software (5). The Gonnet protein weight matrix was used; gap opening and gap extension penalties were 10.0 and 0.1, respectively. Residues in red are identical, green highly similar and blue weakly similar. Five amino acids present in the ligand binding pocket are fully conserved and shaded in yellow. The corresponding amino acids are circled in Fig. 7B.

10 20 30 40 50 60

| | | | | |

WP_284407211110_187 GTLLGSGFALVIAIGFLVAAFGRVQLDHIGGNIKLLSHERIANLLVLQEMKDGINTVARA

WP_248588623110_207 GSMLGIGFTMVIVIGFLVAIYGQLQLRQLSTDIQTLSKKRITNLLLIQEYKDNLNIAARV

WP_198317298110_211 GSMLGVGFTMVIAIGFLVAIYGQSQLRQLSTNIQTLSKERITNLLLIQEYKDNLNIAARV

WP_137715051110_207 GTMLGSGFTIVILIGCFIALFGRVQLTELSNNIQLLSQVRINNLLLMQEVKDNVNIISRV

WP_161451117110_207 GSMLGTGFAMVIVIGFLVAIYGQLQLRQLSTNIQTLSKERITNLLLIQEYKDNLNIAARV

WP_209126666110_211 GSMLGTGFTLVIAIGFLVAIYGQLQLRQLSGDIQSLSKERITNLLLIQEYKDNLNIAARV

WP_121480737110_211 GSMLGTGFAMVIVIGFLVAIYGQVQLRQLSTNIQTLSKERLTNLLLIQEYKDNLNVAARV

WP_198298549110_211 GSMLGVGFTMVIAIGFLVAIYGQSQLRQLSTNIQTLSKERITNLLLIQEYKDNLNIAARV

MCL6405923110_207 GSMLGTGFAMVIVIGFLVAIYGQLQLRQLSTNIQTLSKERITNLLLIQEYKDNLNIAARV

WP_210182408110_207 GSMLGIGFTMVIVIGFLVAIYGQLQLRQLGTDIQTLSQKRITNLLLIQEYKDNMNIAARV

WP_038899989110_207 GSMLGTGFAMVIVIGFLVAIYGQLQLRQLSTNIQTLSKERITNLLLIQEYKDNLNIAARV

WP_198299881110_211 GSMLGVGFTMVIAIGFLVAIYGQSQLRQLSTNIQTLSKERITNLLLIQEYKDNLNIAARV

WP_342699658110_207 GSMLGIGFTMVIVIGFLVAIYGQLQLRQLGTDIQTLSKKRITNLLLIQEYKDNLNIAARV

WP_264090912110_207 GTMLGSGFAIVILIGCLVALFGRMQLAELGNNIQLLSQVRINNLLLMQDVKDNINTISRS

WP_168361165110_207 GSMLGIGFTMVIAIGFLVAIYGQLQLRQLGTDIQTLSKKRITNLLLIQEYKDNMNITARI

WP_226511591110_207 GSMLGIGFTMVIVIGFLVAIYGQLQLRQLGTDIQTLSQKRITNLLLIQEYKDNMNIAARV

WP_242751928110_211 GSMLGVGFTMVIAIGFLVAIYGQSQLRQLSTHIQTLSKERITNLLLIQEYKDNLNIAARV

WP_013315853110_207 GSMLGTGFAMVIVIGFLVAIYGQLQLRQLSTNIQTLSKERITNLLLIQEYKDNLNIAARV

WP_192458598112_207 --MLGTGFAMVIAIGFLVAIYGQIQLKQLSTHIQTLSKERLTELQLIQEYKDNMNITARV

WP_198314307110_211 GSMLGVGFTMVIAIGFLVAIYGQSQLRQLSTNIQTLSKERITNLLLIQEYKDNLNIAARV

WP_038921308110_211 GSMLGTGFAMVIVIGFLVAIYGQMQLRQLSTNIQTLSKERLTNLLLIQEYKDNLNIAARV

WP_406618536110_211 GSMLGTGFTLVIAIGFLVAIYGQLQLRQLSGDIQSLSKERITNLLLIQEYKDNLNIAARV

WP_303313259110_211 GSMLGTGFAMVIVIGFLVAIYGQVQLRQLSTNIQTLSKERLTNLLLIQEYKDNLNVAARV

WP_038914843110_207 GSMLGIGFTMVIAIGFLVAIYGQLQLRQLGTDIQTLSQKRITNLLLIQEYKDNMNIAARV

WP_102802572110_207 GSMLGIGFTMVIAIGFLVAIYGQLQLRQLGTDIQTLSQKRITNLLLIQEYKDNMNIAARV

WP_220177006110_207 GSMLGIGFTMVIAIGFLVAIYGQLQLRQLGTDIQTLSKKRITNLLLIQEYKDNMNITARI

WP_284431376110_187 GTLLGSGFALVIAIGFLVAAFGRVQLDHIGGNIKLLSHERIANLLVLQEMKDGINTVARA

WP_024104118110_211 GSMLGVGFTMVIAIGFLVAIYGQSQLRQLSTNIQTLSKERITNLLLIQEYKDNLNIAARV

MCG8709529110_192 GTMLGSGFALIILIGCLVSLFGRMQLAELGSNIQ-LSQVRINNLLLMQEVKDNINTLSRV

WP_336678565110_211 GSMLGVGFTMVIAIGFLVAIYGQSQLRQLSTNIQTLSKERITNLLLIQEYKDNLNIAARV

WP_253940658110_211 GSMLGTGFAMVIVIGFLVAIYGQLQLRQLSTNIQTLSKERITNLLLIQEYKDNLNIAARV

WP_171861319110_207 GSMLGTGFAMVIVIGFLVAIYGQLQLRQLSTNIQTLSKERITNLLLIQEYKDNLNIAARV

WP_284414662110_190 GTLLGSGFALVIAIGFLVAAFGRIQLDQVGGNIKLLSHDRVATLLLLQEMKDGINTAARA

NMN9323418_205 GTMLGSGFAIVILIGCLVALFGRMQLAELGNNIQLLSQVRINNLLLMQDVKDNINSISRS

WP_284414646110_187 GTLLGSGFALVIAIGFLVAAFGRVQLDHIGGNIKLLSHERIANLLVLQEMKDGINTVARA

WP_198299430110_211 GSMLGVGFTMVIAIGFLVAIYGQSQLRQLSTNIQTLSKERITNLLLIQEYKDNLNIAARV

WP_148042947110_207 GSMLGSGFALVIVIGFLVAIYGQIQLRQLSTNIQTLSKEQLTNLLLIQEYKDNLNVAARV

RLM2168211_184 --MLGSGFALIILIGCLVSIFGRMQLADLGGNIQ-LSQVRINNLLLMQEVKDNINTMSRV

WP_033568988110_207 GSMLGSGFALVIVIGFLVAIYGQIQLRQLSTNIQTLSKEQLTNLLLIQEYKDNLNVAARV

WP_171850212110_207 GSMLGTGFAMVIVIGFLVAIYGQLQLRQLSTNIQTLSKERITNLLLIQEYKDNLNIAARV

WP_019845382110_207 GSMLGIGFTMVIAIGFLVAIYGQLQLRQLGTDIQTLSQKRITNLLLIQEYKDNMNIAARV

WP_272550727110_184 GTLLGSGFALVIAIGFLVAAFGRFQLDHVGGNIKLLSHERIANLLVLQEMKDGINTVARA

WP_146410942111_190 -SMLGTGFAMVIAIGFLVAIYGQLQLRHLSTDIQMLSKDRITNLLLIQEYKDNMNIVARV

WP_17184960718_195 GSMLGTGFAMVIVIGFLVAIYGQLQLRQLSTNIQTLSKERITNLLLIQEYKDNLNIAARV

WP_208228909110_195 GTMLGAGFALIILIGCLVSLFGRMQLADLSGNIQ-LSQVRINNLLLMQEVKDNINTMSRV

WP_113865726110_207 GTMLGSGFAIVILIGCLVALFGRMQLAELGNNIQLLSQVRINNLLLMQDVKDNINSISRS

WP_161132035110_211 GSMLGVGFTMVIAIGFLVAIYGQSQLRQLSTHIQTLSKERITNLLLIQEYKDNLNIAARV

WP_201743379110_207 GSMLGSGFALVIVIGFLVAIYGQIQLRQLSTNIQTLSKEQLTNLLLIQEYKDNLNVAARV

WP_038664436110_211 GSMLGTGFAMVIVIGFLVAIYGQVQLRQLSTNIQTLSKERLTNLLLIQEYKDNLNVAARV

WP_100850303110_211 GSMLGTGFTLVIAIGFLVAIYGQLQLRQLSGDIQSLSKERITNLLLIQEYKDNLNIAARV

WP_012886618110_207 GSMLGIGFTMVIVIGFLVAIYGQLQLRQLGTDIQTLSQKRITNLLLIQEYKDNMNIAARV

WP_198314835110_211 GSMLGVGFTMVIAIGFLVAIYGQSQLRQLSTNIQTLSKERITNLLLIQEYKDNLNIAARV

MDQ5892624110_207 GKMLGIGFTLIIAIGFLVAVFGRIQLDKLGENIQLLSQVRITNLMMMKEFKDNINTNAIV

WP_038917453110_211 GSMLGTGFTLVIAIGFLVAIYGQLQLRQLSGDIQSLSKERITNLLLIQEYKDNLNIAARV

WP_224062336110_207 GSMLGTGFAMVIVIGFLVAIYGQLQLRQLSTNIQTLSKERITNLLLIQEYKDNLNIAARV

WP_121589257110_195 GTMLGSGFALIILIGCLVSIFGRMQLADLGGNIQ-LSQVRINNLLLMQEVKDNINTMSRV

WP_048636656110_195 GTMLGSGFALIILIGCLVSIFGRMQLADLGGNIQ-LSQVRINNLLLMQEVKDNINTMSRV

WP_210173983110_207 GSMLGIGFTMVIVIGFLVAIYGQLQLRQLGTDIQTLSQKRITNLLLIQEYKDNLNIAARV

WP_253000914110_207 GSMLGIGFTMVIVIGFLVAIYGQLQLRQLSTDIQTLSKKRITNLLLIQEYKDNLNIAARV

WP_284603643110_207 GSMLGTGFAMVIVIGFLVAIYGQLQLRQLSTNIQTLSKERITNLLLIQEYKDNLNIAARV

WP_198298864110_211 GSMLGVGFTMVIAIGFLVAIYGQSQLRQLSTNIQTLSKERITNLLLIQEYKDNLNIAARV

WP_172290490110_206 GTMLGSGFAVIILIGFVVSLFGRMQLAELGGNIQ-LSQVRINNLLLMQEVKDNINTLSRV

MEQ4512139110_207 GSMLGIGFTMVIAIGFLVAIYGQLQLRQLGTDIQTLSQKRITNLLLIQEYKDNMNIAARV

WP_023641026110_207 GSMLGIGFTMVIAIGFLVAIYGQLQLRQLGTDIQTLSQKRITNLLLIQEYKDNMNIAARV

WP_336684953110_207 GSMLGTGFAMVIVIGFLVAIYGQLQLRQLSTNIQTLSKERITNLLLIQEYKDNLNIAARV

WP_168366716110_207 GSMLGIGFTMVIVIGFLVAIYGQLQLRQLSTDIQTLSKKRITNLLLIQEYKDNLNIAARV

WP_150436034110_196 GTMLGSGFALLILIGFLVAAFGRVQLTHLGDDIQLLSKVRINNLLMMQEVKDNVNTVARA

WP_198314030110_211 GSMLGVGFTMVIAIGFLVAIYGQSQLRQLSTNIQTLSKERITNLLLIQEYKDNLNIAARV

WP_263064027110_211 GSMLGTGFAMVIVIGFLVAIYGQMQLRQLSTNIQTLSKERLTNLLLIQEYKDNLNIAARV

WP_049853775110_211 GSMLGTGFAMVIVIGFLVAIYGQVQLRQLSTNIQTLSKERLTNLLLIQEYKDNLNVAARV

WP_015855510112_207 --MLGTGFAMVIAIGFLVAIYGQIQLKQLSTHIQTLSKERLTELQLIQEYKDNMNITARV

WP_175537819110_190 GTLLGSGFALVIAIGFLVAAFGRVQLDQVGSNIKLLSHDRVANLLLLQEMKDGLNTVARA

WP_219952136110_207 GSMLGIGFTMVIAIGFLVAIYGQLQLRQLGTDIQTLSKKRITNLLLIQEYKDNMNITARI

WP_272566864110_187 GTLLGSGFALVIAIGFLVAAFGRVQLDHIGGNIKLLSHERIANLLVLQEMKDGINTVARA

WP_284402436110_187 GTLLGSGFALVIAIGFLVAAFGRVQLDHIGGNIKLLSHERIANLLVLQEMKDGINTVARA

WP_033577122110_207 GSMLATGFTMVIVIGFLVAIYGQSQLRQLSVDIQTLSKERLTKLLLIQEYKDNLNIAARV

WP_226068163110_207 GSMLGIGFTMVIAIGFLVAIYGQLQLRQLGTDIQTLSQKRITNLLLIQEYKDNMNIAARV

WP_022635338110_211 GSMLGTGFTMVIVIGFLVAIYGQMQLRQLSTNIQTLSKERLTNLLLIQEYKDNLNIAARV

WP_253005091110_207 GSMLGIGFTMVIAIGFLVAIYGQLQLRQLGTDIQTLSKKRITNLLLIQEYKDNMNIAARV

WP_198317810110_211 GSMLGVGFTMVIAIGFLVAIYGQSQLRQLSTNIQTLSKERITNLLLIQEYKDNLNIAARV

WP_284426831110_187 GTLLGSGFALVIAIGFLVAAFGRVQLDHIGGNIKLLSHERIANLLVLQEMKDGINTVARA

WP_239788193110_211 GSMLGTGFTLVIAIGFLVAIYGQLQLRQLSGDIQSLSKERITNLLLIQEYKDNLNIAARV

WP_175637768110_211 GSMLGTGFAMVIVIGFLVAIYGQLQLRQLSTNIQTLSKERITNLLLIQEYKDNLNIAARV

WP_168641181112_190 --MLGTGFAMVIAIGFLVAIYGQLQLRHLSTDIQMLSKDRITNLLLIQEYKDNLNITARV

PacG GKMLGTGFTLIIVIGFLVAILGRVQLERLGGNIQLLSQIRITNLLLMQEVKDNVNDTARA

WP_336700672110_207 GSMLATGFTMVIVIGFLVAIYGQSQLRQLSVDIQTLSKERLTNLLLIQEYKDNLNIAARV

WP_121574097110_189 GTMLGSGFALIILIGCLVALFGRVQLTELGGNIQ-LSQVRINNLLLMQEVKDNINTMSRI

WP_317355727110_187 GTLLGSGFALVIAIGFLVAAFGRVQLDHIGGNIKLLSHERIANLLVLQEMKDGINTVARA

WP_316393554110_211 GSMLGTGFAMVIVIGFLVAIYGQMQLRQLSTNIQTLSKERLTNLLLIQEYKDNLNIAARV

WP_407322000110_207 GSMLGIGFTMVIVIGFLVAIYGQLQLRQLGTDIQTLSKKRITNLLLIQEYKDNLNIAARV

WP_374069696110_207 GSMLGTGFAMVIVIGFLVAIYGQLQLRQLSTNIQTLSKERITNLLLIQEYKDNLNIAARV

ATA2706911_184 --MLGSGFALIILIGCLVSIFGRMQLADLGGNIQ-LSQVRINNLLLMQEVKDNINTMSRV

WP_038917000110_207 GSMLGIGFTMVIAIGFLVAIYGQLQLRQLGTDIQTLSQKRITNLLLIQEYKDNMNIAARV

WP_143839830110_207 GSMLATGFTMVIVIGFLVAIYGQSQLRQLSVDIQTLSKERLTNLLLIQEYKDNLNIAARV

WP_271465838110_207 GAMLGMGFAVVIAIGFMVAAYGRIQLDQVGSNVQALSKDRLGTLLVLQEVKDNINIVARA

WP_038909929110_207 GSMLGTGFAMVIVIGFLVAIYGQLQLRQLSTNIQTLSKERITNLLLIQEYKDNLNIAARV

WP_038909309110_207 GSMLGIGFTMVIVIGFLVAIYGQLQLRQLGTDIQTLSKKRITNLLLIQEYKDNLNIAARV

WP_042867702111_190 -SMLGTGFAMVIAIGFLVAIYGQLQLRHLSTDIQMLSKDRITNLLLIQEYKDNMNIVARV

NPE69876110_207 GSMLGTGFAMVIVIGFLVAIYGQLQLRQLSTNIQTLSKERITNLLLIQEYKDNLNIAARV

WP_039693058110_211 GSMLGTGFTLVIAIGFLVAIYGQLQLRQLSGDIQSLSKERITNLLLIQEYKDNLNIAARV

WP_226099784110_207 GSMLGIGFTMVIVIGFLVAIYGQLQLRQLGTDIQTLSKKRITNLLLIQEYKDNLNIAARV

70 80 90 100 110 120

| | | | | |

WP_284407211110_187 ARNIALLEDIPQMADEKRRIDKAQARNAELLSQLNQRMTSPEAKALVAQIGQARPAYVEA

WP_248588623110_207 IRNIVLLSEPQQMAVEKQRLDNMAPRNNEIIATLHNVATTPEQKALIAQLDQNRPIYEEA

WP_198317298110_211 IRNIALLSDHQQMTEEKQRIDNMAPRNDDIIATLHKVAVTPEQKALMAQLDQNRPAYQDA

WP_137715051110_207 VRNIALMDNPQEMEKEKKRVDERLSINTKLLNTLRTTAVSPEARQRIEALIQHSPSYRDA

WP_161451117110_207 IRNIALLSDHQQMTVEKQRLDNMAPRNDDIIAILHKVAVTPEQQALIAQLDQNRPAYQDA

WP_209126666110_211 IRNIALLSDPKQMATEKQRIDAIIPRNSEIIAILRKTSVTPEQNALLDQLEQKRPAYLEA

WP_121480737110_211 IRNIALLSDQQQMAVEKQRIDAIIPRNSDIITILRKASETAEQNALLDQLEQKRPAYLEA

WP_198298549110_211 IRNIALLSDHQQMTEEKQRIDNMAPRNDDIIATLHKVAVTPEQKALMAQLDQNRPAYQDA

MCL6405923110_207 IRNIALLSDHQQMTVEKQRLDNMAPRNDDIIAILHKVAVTPEQQALIAQLDQNRPVYEDA

WP_210182408110_207 IRNIVLLSEPQQMAIEKQRLDNMAPRNNEIIATLHNAATTAEQKALIAQLDQYRPIYEEA

WP_038899989110_207 IRNIALLSDHQQMIVEKQRLDNMAPRNDDIIAILHKVAVTPEQQALIAQLDQNRPAYQDA

WP_198299881110_211 IRNIALLSDHQQMTEEKQRIDNMAPRNDDIIATLHKVAVTPEQKALMAQLDQNRPAYQDA

WP_342699658110_207 IRNIVLLSEPQQMAVEKQRLDNMVPRNDEIIATLRNAATTPEQKALIAQLDQNRPIYEEA

WP_264090912110_207 VRNITLMENNQEKESEKNRIDELLSRNAEHLNTLRNTAVSPEARQLVQALAQLSPTYRET

WP_168361165110_207 IRNIALLSEPQQMAVEKQRLDNMAPRNNEIIAILHNVATTAEQKALIAQLDQYRPIYEEA

WP_226511591110_207 IRNIVLLSEPQQMAIEKQRLDNMAPRNNEIIATLHNAATTAEQKALIAQLDQYRPIYEEA

WP_242751928110_211 IRNIALLSDHQQMTEEKQRIDNMAPRNDAIIAALHKVAVTPEQKALMAQLDQNRPAYQDA

WP_013315853110_207 IRNIALLSDHQQMTFEKQRLDNMAPRNDDIIAILHKVAVTPEQQALIAQLDQNRPVYEDA

WP_192458598112_207 IRNIALLTDQQQMLIEKQRIDTIIPRNSDLLVLLRKTSTTAEQNSLLDQLEQKRPAYLEA

WP_198314307110_211 IRNIALLSDHQQMTEEKQRIDNMAPRNDDIIATLHKVAVTPEQKALMAQLDQNRPAYQDA

WP_038921308110_211 IRNIALLSDQQQMAVEKQRIDAIIPRNSDIIALLRKASETAEQNALLDQLEQKRPAYLEA

WP_406618536110_211 IRNIALLSDPKQMTTEKQRIDAIIPRNSEIIAILRKTSVTPEQNALLDQLEQKRPAYLEA

WP_303313259110_211 IRNIALLSDQQQMAVEKQRIDAIIPRNSDIITILRKASETAEQNALLDQLEQKRPAYLEA

WP_038914843110_207 IRNIVLLSEPQQMAVEKQRLDNMAPRNNEIIATLHSAANTAEQKALIAQLDQYRPIYEEA

WP_102802572110_207 IRNIVLLSEPQQMAVEKQRLDNMAPRNNEIIATLHSAATTAEQKALIAQLDQYRPIYEEA

WP_220177006110_207 IRNIALLSEPQQMAVEKQRLDNMAPRNNEIIATLHNVATTAEQKALIAQLDQYRPIYEEA

WP_284431376110_187 ARNIALLEDLPQMADEKRRIDKAQARNAELLSQLNQRMTSPEAKALVAQIGQARPAYVEA

WP_024104118110_211 IRNIALLSDHQQMTEEKQRIDNMAPRNDDIIATLHKVAVTPEQKALMAQLDQNRPAYQDA

MCG8709529110_192 IRNIALMDDRQEMEGERNRIIQLQARNDELFTQIRQRTISPEARTRTQNLEKLIPAYLET

WP_336678565110_211 IRNIALLSDHQQMTEEKQRIDNMAPRNDDIIATLHKVAVTPEQKALMAQLDQNRPAYQDA

WP_253940658110_211 IRNIALLSDHQQMIVEKQRLDNMAPRNDDIIAILHKVAVTPEQQALIAQLDQNRPAYQDA

WP_171861319110_207 IRNIALLSDHQQMTVEKQRLDNMAPRNDDIIAILHKVAVTPEQQALIAQLDQNRPAYQDA

WP_284414662110_190 ARNIALLEDIAQMADEKRRIEQTQARNVELLGQLGPRMSTPEAGALFAKIGEARPAYRDA

NMN9323418_205 VRNIALTENRQEMESEKNRIDELLSRNAKHLDTLRNTAVSPEARQRVQALAQLSPTYRET

WP_284414646110_187 ARNIALLEDLPQMADEKRRIDKAQARNAELLSQLNQRMTSPEAKALVAQIGQARPAYVEA

WP_198299430110_211 IRNIALLSDHQQMTEEKQRIDNMAPRNDDIIATLHKVAVTPEQKALMAQLDQNRPAYQDA

WP_148042947110_207 IRNIALLSDQQQMATEKQRIDTITPRNSEIIAILRKTSGTAEQNALLDQLEQKRPAYLEA

RLM2168211_184 IRNIALMDNRQEMEGEQKRIVELRERNSELFAQIRQRTISPEARIRTQNLEKLIPSYLET

WP_033568988110_207 IRNIALLSDQQQMATEKQRIDTITPRNSEIIAILRKTSGTAEQNALLDQLEQKRPAYLEA

WP_171850212110_207 IRNIALLSDHQQMTVEKQRLDNMAPRNDDIIAILHKVAVTPEQQALIAQLDQNRPVYEDA

WP_019845382110_207 IRNIVLLSEPQQMAVEKQRLDNMAPRNNEIIATLHSAANTAEQKALIAQLDQYRPIYEEA

WP_272550727110_184 ARNIALLEDIPQMTDEKRRIDKAQARNAELLSHLSQQMTSPEAKALIAQIGQARPAYVEA

WP_146410942111_190 VRNIVLLSDSQQMVVEKQRIDSTASRNDEIIAMLHKVAVTPEQKTLLAQLEQSRPVYQEA

WP_17184960718_195 IRNIALLSDHQQMTVEKQRLDNMAPRNDDIIAILHKVAVTPEQQALIAQLDQNRPAYQDA

WP_208228909110_195 IRNIALLEDRQEMEGEQKRIVQLQARNSELFTQIRERTISPEARTRTQNLEKLIPSYLET

WP_113865726110_207 VRNIALTENRQEMESEKNRIDELLSRNAKHLDTLRNTAVSPEARQRVQALAQLSPTYRET

WP_161132035110_211 IRNIALLSDHQQMTEEKQRIDNMAPRNDAIIAALHKVAVTPEQKALMAQLDQNRPAYQDA

WP_201743379110_207 IRNIALLSDQQQMATEKQRIDTITPRNSEIIAILRKTSGTAEQNALLDQLEQKRPAYLEA

WP_038664436110_211 IRNIALLSDQQQMAVEKQRIDAIIPRNSDIITILRKASETAEQNALLDQLEQKRPAYLEA

WP_100850303110_211 IRNIALLSDPKQMATEKQRIDAIIPRNSEIIAILRKTSVTPEQNALLDQLEQKRPAYLEA

WP_012886618110_207 IRNIVLLSEPQQMAIEKQRLDNMAPRNNEIIATLHNAATTAEQKALIAQLDQYRPIYEEA

WP_198314835110_211 IRNIALLSDHQQMTEEKQRIDNMAPRNDDIIATLHKVAVTPEQKALMAQLDQNRPAYQDA

MDQ5892624110_207 IRNLTMREDSRKMQEEKTHIDNMIARNNALLAKIRDRAIDQHSQELIAALDRVRPTYNDS

WP_038917453110_211 IRNIALLSDPKQMTTEKQRIDAIIPRNSEIIAILRKTSVTPEQNALLDQLEQKRPAYLEA

WP_224062336110_207 IRNIALLSDHQQMTVEKQRLDNMAPRNDDIIAILHKVAVTPEQQALIAQLDQNRPVYEDA

WP_121589257110_195 IRNIALMDNRQEMEGEQKRIVELRERNSELFAQIRQRTISPEARIRTQNLEKLIPSYLET

WP_048636656110_195 IRNIALMDNRQEMEGEQKRIVELRERNSELFAQIRQRTISPEARIRTQNLEKLIPSYLET

WP_210173983110_207 IRNIVLLSEPQQMALEKQRLDNMAPRNNEIIATLHNVANTPEQKAMIAQLDQYRPIYEEA

WP_253000914110_207 IRNIVLLSEPQQMAVEKQRLDNMVPRNNEIIATLHNVATTPEQKALIAQLDQNRPIYEEA

WP_284603643110_207 IRNIALLSDHQQMTVEKQRLDNMAPRNDDIIATLHKVAVTPEQQALIAQLDQNRPVYEDA

WP_198298864110_211 IRNIALLSDHQQMTEEKQRIDNMAPRNDDIIATLHKVAVTPEQKALMAQLDQNRPAYQDA

WP_172290490110_206 IRNIALMDDRQEMEEQRQRIVRLQARNAELFTQIRENTISPEARTRTQNLEKLIPSYIET

MEQ4512139110_207 IRNIVLLSEPQQMAVEKQRLDNMAPRNNEIIATLHSAATTAEQKALIAQLDQYRPIYEEA

WP_023641026110_207 IRNIVLLSEPQQMAVEKQRLDNMAPRNNEIIATLHSAATTAEQKALIAQLDQYRPIYEEA

WP_336684953110_207 IRNIALLSDHQQMTVEKQRLDNMAPRNDDIIAILHKVAVTPEQQALIAQLDQNRPAYQDA

WP_168366716110_207 IRNIVLLSEPQQMAVEKQRLDNMAPRNNEIIATLHNVATTPEQKALIAQLDQNRPIYEEA

WP_150436034110_196 IRDLLLLDDPAAMETEKQKIEALLQQNTQLLSKIQAGTVSPEAQARTEKLVRMRIPYNNI

WP_198314030110_211 IRNIALLSDHQQMTEEKQRIDNMAPRNDDIIATLHKVAVTPEQKALMAQLDQNRPAYQDA

WP_263064027110_211 IRNIALLSDQQQMAVEKQRIDAIIPRNSDIIALLRKASETAEQSALLDQLEQKRPAYLEA

WP_049853775110_211 IRNIALLSDQQQMAVEKQRIDAIIPRNSDIITILRKASETAEQNALLDQLEQKRPAYLEA

WP_015855510112_207 IRNIALLTDQQQMLIEKQRIDTIIPRNSDLLVLLRKTSTTAEQNSLLDQLEQKRPAYLDA

WP_175537819110_190 ARNIALLEDPTQMAEEKRRIEQTQARNLELIGQLNTRMSSPEARALFAKIGEARPAYVAA

WP_219952136110_207 IRNIALLSEPQQMAVEKQRLDNMAPRNNEIIAILHNVATTAEQKALIAQLDQYRPIYEEA

WP_272566864110_187 ARNIALLEDIPQMADEKRRIDKAQARNAELLSQLNQRMTSPEAKALVAQIGQARPAYVEA

WP_284402436110_187 ARNIALLEDIPQMADEKRRIDKAQARNAELLSQLNQRMTSPEAKALVAQIGQARPAYVEA

WP_033577122110_207 IRNIALLADPQQMAVEKQRLDNMAPRNDAILATLHNVAVTPEQKALIAQLDQNRPVYEDA

WP_226068163110_207 IRNIVLLSEPQQMAVEKQRLDNMAPRNNEIIATLHSAATTAEQKALIAQLDQYRPIYEEA

WP_022635338110_211 IRNIALLSDQQQMAVEKQRLDNMAPRNDDIIATLHKVAVTPEQKALMAQLDQNRPAYQDA

WP_253005091110_207 IRNIVLLSEPQQMAVEKQRLDNMAPRNNEIIAILHNVATTAEQKALIAQLDQYRPIYEDA

WP_198317810110_211 IRNIALLSDHQQMTEEKQRIDNMAPRNDDIIATLHKVAVTPEQKALMAQLDQNRPAYQDA

WP_284426831110_187 ARNIALLEDIPQMADEKRRIDKAQARNAELLSQLNQRMTSPEAKALVAQIGQARPAYVEA

WP_239788193110_211 IRNIALLSDPKQMATEKQRIDAIIPRNSEIIAILRKTSVTPEQNALLDQLEQKRPAYLEA

WP_175637768110_211 IRNIALLSDHQQMTVEKQRLDNMAPRNDDIIAILHKVAVTPEQQALIAQLDQNRPVYEDA

WP_168641181112_190 VRNIVLLSDSQQMRVEKQRIDGVESRNDEIIAMLHKVAVTPEQKTLLTQLEQSRPVYQEA

PacG IRNMALLNDQQQMKTEKERIEKSIARNNDLLAQIRKNTVSSETKVQVATLEQALPAYINN

WP_336700672110_207 IRNIALLADPQQMAVEKQRLDNMSPRNNAILATLHNVAVTPEQKALIAQLDQNRPVYEDA

WP_121574097110_189 IRNIALMDDRQEMEEEEKRIVKLLARNSELFSQIKERTVSPEARARTQALEALIPSYRAT

WP_317355727110_187 ARNIALLEDLPQMADEKRRIDKAQARNAELLSQLNQRMTSPEAKALVAQIGQARPAYVEA

WP_316393554110_211 IRNIALLSDQQQMAVEKQRIDAIIPRNSDIIAMLRKASETVEQSALLDQLEQKRPAYLEA

WP_407322000110_207 IRNIVLLSEPQQMAVEKQRLDNMVPRNDEIIATLRNAATTPEQKALIAQLDQNRPIYEEA

WP_374069696110_207 IRNIALLSDHQQMTVEKQRLDNMAPRNDDIIAILHKVAVTPEQQALIAQLDQNRPAYQDA

ATA2706911_184 IRNIALMDNRQEMEGEQKRIVELRERNSELFAQIRQRTISPEARIRTQNLEKLIPSYLET

WP_038917000110_207 IRNIVLLSEPQQMAVEKQRLDNMAPRNNEIIATLHSAANTAEQKALIAQLDQYRPIYEEA

WP_143839830110_207 IRNIALLADPQQMAVEKQRLDNMAPRNDAILATLHNVAVTPEQKALIAQLDQNRPVYEDA

WP_271465838110_207 VRNLALIEDPQTMNQEVARLNDVIQRTSQVMGDLQQRVRSPEARAFMARIAEVRPAFLDA

WP_038909929110_207 IRNIALLSDHQQMIVEKQRLDNMAPRNDDIIAILHKVAVTPEQQALIAQLDQNRPVYEDA

WP_038909309110_207 IRNIVLLSEPQQMALEKQRLDNMAPRNNEIIATLHNVANTPEQKAMIAQLDQYRPIYEEA

WP_042867702111_190 VRNIVLLSDSQQMVVEKQRIDSTASRNDEIIAMLHKVAVTPEQKTLLAQLEQSRPVYQEA

NPE69876110_207 IRNIALLSDHQQMIVEKQRLDNMAPRNDDIIAILHKVAVTPEQQALIAQLDQNRPAYQDA

WP_039693058110_211 IRNIALLSDPKQMATEKQRIDAIIPRNSEIIAILRKTSVTPEQNALLDQLEQKRPAYLEA

WP_226099784110_207 IRNIVLLSEPQQMALEKQRLDNMAPRNNEIIATLHNVANTPEQKAMIVQLDQYRPIYEEA

130 140 150 160 170 180

| | | | | |

WP_284407211110_187 LNKAVDLGLANQNDQAREQLLGPVRPVQAAYFKALDDLVDYQKAATVRTADESEKDAV--

WP_248588623110_207 VRKALEKGLANKDDEARELIITDVRRAQDSLFKIIDTMLAYQKNATTVTADNSQSQAVNA

WP_198317298110_211 VRKAIDKGLENKDDEARDLILNDVRRSQDALFKAIDTMLTYQKSATTATADDSESQANGA

WP_137715051110_207 LYKAIDLGMSGQKAETRAVVFGEMRVAQTNVFNALDEMVAYQKSLTVEAANTSAQQADTS

WP_161451117110_207 VGRAIEKGLENKDDDARNLILTDVRRAQDNMFKAIDSLLAYQKSATSTTADDSESRASNA

WP_209126666110_211 VRKTIELGLSNQPDNARNLILGDVRNAQDALFKVIETMLTYQKNATSTAADSSESQADNA

WP_121480737110_211 VRKAIELGLSNQSEKARSLILGDVRNAQDALFKVIETMLTYQKNATSTAADSSESQADNA

WP_198298549110_211 VRKAIDKGLENKDDEARDLILNDVRRSQDALFKAIDTMLTYQKSATTATADDSESQANGA

MCL6405923110_207 VGRAIEKGLENKDDDARNLILTDVRRAQENMFKVIDSLLAYQKSATSTTADDSESRASNA

WP_210182408110_207 VKKALEKGLANKDDEAREFILTDVRRAQDSLFKVIDTMLAYQKNATTVTAENSQSQAVNG

WP_038899989110_207 VGRAIEKGLENKDDDARNLILTDVRRAQDNMFKAIDSLLAYQKSATSTTADDSESRASNA

WP_198299881110_211 VRKAIDKGLENKDDEARDLILNDVRRSQDALFKAIDTMLTYQKSATTATADDSESQANGA

WP_342699658110_207 LKKALDKGLANKDDEARELIITDVRRAQDSLFKVIDTMLAYQKNATAVTADNSQSQAVNA

WP_264090912110_207 LNKTINLGISGQMQEARAMLFGEMRDAQANVFSALDEMVTYQKSLTVETANISERQAITA

WP_168361165110_207 VKKALEKGLANKNDEARELILTEVRRAQDSLFKVIDTMLAYQKNATAVTADNSQSQAVNA

WP_226511591110_207 VKKALEKGLANKDDEAREFILTDVRRAQDSLFKVIDTMLAYQKNATTVTAENSQSQAVNA

WP_242751928110_211 VRKAVEKGLENKDDEARELILNDVRRSQDALFKAIDTMLTYQKSATTATADDSESQANGA

WP_013315853110_207 VGRAIEKGLENKDDDARNLILTDVRRAQENMFKVIDSLLAYQKSATSTTADDSESRASNA

WP_192458598112_207 IRKTIEFGLSNQPDNAKNLILGEVRNTQDTLFKVIDSLLNYQKSVTNATADNSESQANNA

WP_198314307110_211 VRKAIDKGLENKDDEARDLILNDVRRSQDALFKAIDTMLTYQKSATTATADDSESQANGA

WP_038921308110_211 VRKTIELGLSNQSDKARSLILSDVRNAQDSLFKVIETMLTYQKNSTSATADDSESQANSA

WP_406618536110_211 VRKTIELGLSNQPDNARNLILGDVRSTQDALFKVIESMLTYQKNATSTAADSSESQADSA

WP_303313259110_211 VRKTIELGLSNQSEKARSLILGDVRNAQDALFKVIETMLTYQKNATSTAADSSESQADNA

WP_038914843110_207 IKKALEKGLANKDDEAREFIITDVRRAQDSLFKVIDTMLAYQKNATAVTADNSQSQAVNA

WP_102802572110_207 IKKAIEKGLANKDDEAREFIITDVRRAQDSLFKVIDTMLAYQKNATAVTADNSQSQAVNA

WP_220177006110_207 VKKALDKGLANKNDEAREFILTDVRRAQDSLFKVIDTMLAYQKNATAVTADNSQSQAVNA

WP_284431376110_187 LNKAVDLGLANQNDQAREQLLGPVRPVQAAYFKALDDLVDYQKAATVRTADESEKDAV--

WP_024104118110_211 VRKAIDKGLENKDDEARDLILNDVRRSQDALFKAIDTMLTYQKSATTATADDSESQANGA

MCG8709529110_192 VHRSVDAGMSGDSTQIRTVLFGDLRDAQNGIFNALDAMIDYQTTLTIETAEKSQQRATSA

WP_336678565110_211 VRKAIDKGLENKDDEARDLILNDVRRSQDALFKAIDTMLTYQKSATTATADDSESQANGA

WP_253940658110_211 VGRAIEKGLENKDDDARNLILTDVRRAQDNMFKAIDSLLAYQKSATSTTADDSESRASNA

WP_171861319110_207 VGRAIEKGLENKDDDARNLILTDVRRAQDNMFKAIDSLLAYQKSATSATADDSESRASNA

WP_284414662110_190 LNKAVDLGMANQNEQARAQLLGPVRPTQAAYFKALDDVIAYQKAATASTAELSEQSAASA

NMN9323418_205 LNKAINSGLSGQMQEARAILFGEMRDAQANVFSALDEMVTYQKGLTVETANTSERQAITA

WP_284414646110_187 LNKAVDLGLANQNDQAREQLLGPVRPVQAAYFKALDDLVDYQKAATVRTADESEKDAV--

WP_198299430110_211 VRKAIDKGLENKDDEARDLILNDVRRSQDALFKAIDTMLTYQKSATTATADDSESQANGA

WP_148042947110_207 AKKAIELGLSNQPDKARSLILSDVRNAQDALFKVIETMLTYQKKATSTAADNSESQAASA

RLM2168211_184 VNKSIDTGMSGNIEETRGMLFGELRDAQNGIFDALDAMIDYQTTLTIETANASQHQATSA

WP_033568988110_207 AKKAIELGLSSQPDKARNLILSDVRNAQDALFKVIETMLTYQKKATSTAADNSESQAASA

WP_171850212110_207 VGRAIEKGLENKDDDARNLILTDVRRAQENMFKAIDSLLAYQKSATSTTADDSESRASNA

WP_019845382110_207 IKKALEKGLANKDDEAREFIITDVRRAQDSLFKVIDTMLAYQKNATAVTADNSQSQAVNA

WP_272550727110_184 LNKAVDMGLANQNDQAREQLLGPVRPVQAAYFKALDDLIDYQKGATVRTADESEK-----

WP_146410942111_190 LRRAIEKGMENKDDEARNLILTEVRRTQETLFKIIDTMLLYQKSATTTTANNSESQASNA

WP_17184960718_195 VGRAIEKGLENKDDDARNLILTDVRRAQDNMFKAIDSLLAYQKSATSTTADDSESRASNA

WP_208228909110_195 VKKSIDAGMSGNIAQARAMLFGELRDAQNGIFDALDAMIDYQTSLTIETANASQHQATTA

WP_113865726110_207 LNKAINSGLSGQMQEARAILFGEMRDAQANVFSALDEMVTYQKGLTVETANTSERQAITA

WP_161132035110_211 VRKAIDKGLENKDDEARELILNDVRRSQDALFKAIDTMLTYQKSATTATADDSESQANGA

WP_201743379110_207 VKKAIELGLSNQPDKARSLILSDVRNAQDALFKVIETMLTYQKKATSTAADNSESQAASA

WP_038664436110_211 VRKTIELGLSNQPDNARNLILSDVRNTQDALFKVIESMLTYQKNATSTAADSSESQADSA

WP_100850303110_211 VRKTIELGLSNQPDNARNLILGDVRSTQDALFKVIENMLTYQKNATSTAADSSESQADNA

WP_012886618110_207 VKKALEKGLANKDDEAREFILTDVRRAQDSLFKVIDTMLAYQKNATTVTAENSQSQAVNA

WP_198314835110_211 VRKAIDKGLENKDDEARDLILNDVRRSQDALFKAIDTMLTYQKSATTATADDSESQANGA

MDQ5892624110_207 ITSAIALVIAHRNKEAQDLLLTDVQAKQDIVFSALNDMVSWQEKITVDAANQSLKNATRA

WP_038917453110_211 VRKAIELGLSNQSEKARSLILGDVRNAQDALFKVIETMLTYQKNATSTAADSSESQADNA

WP_224062336110_207 VGRAIEKGLENKDDDARNLILTDVRRAQENMFKAIDSLLAYQKSATSTTADDSESRASNA

WP_121589257110_195 VNKSIDTGMSGNIEETRGMLFGELRDAQNGIFDALDAMIDYQTTLTIETANASQHQATSA

WP_048636656110_195 VNKSIDTGMSGNIEETRGMLFGELRDAQNGIFDALDAMIDYQTTLTIETANASQHQATSA

WP_210173983110_207 LRKALEKGLENKDDEARELIITDVRRAQDLLFKIIDTMLAYQKNATTVTADNSQSQAVNA

WP_253000914110_207 VKKALEKGLANKDDEARELIITDVRRAQDSLFKIIDTMLAYQKNATTVTADNSQSQAVNA

WP_284603643110_207 VGRAIEKGLENKDDDARNLILTDVRRAQENMFKVIDSLLAYQKSATSTTADDSESRASNA

WP_198298864110_211 VRKAIDKGLENKDDEARDLILNDVRRSQDALFKAIDTMLTYQKSATTATADDSESQANGA

WP_172290490110_206 VNKSISVGMSGQLEETRNILFGELQDAQDGIFNALGEMVTYQATLTIDTANASEHQARTA

MEQ4512139110_207 IKKALEKGLANKDDEAREFIITDVRRAQDSLFKVIDTMLAYQKNATAVTADNSQSQAVNA

WP_023641026110_207 IKKAIEKGLANKDDEAREFIITDVRRAQDSLFKVIDTMLAYQKNATAVTADNSQSQAVNA

WP_336684953110_207 VGRAIEKGLENKDDDARNLILTDVRRAQDNMFKAIDSLLAYQKSATSTTADDSESRASNA

WP_168366716110_207 VKKALEKGLANKDDEARELIITDVRRAQDSLFKIIDTMLAYQKNATTVTADNSQSQAVNA

WP_150436034110_196 LLKTIAYGQSGQNNEARHMLFGDLRQAQDEVFKALDDMVALQETLTVETANASVNRAGSA

WP_198314030110_211 VRKAIDKGLENKDDEARDLILNDVRRSQDALFKAIDTMLTYQKSATTATADDSESQANGA

WP_263064027110_211 VRKTIELGLSNQSDKARSLILSDVRNAQDSLFKVIETMLTYQKNSTSATADDSESQANSA

WP_049853775110_211 VRKAIELGLSNQSEKARSLILGDVRNAQDALFKVIETMLTYQKNATSTAADSSESQADNA

WP_015855510112_207 IGKTIEFGLSNQPDKAKNLILGEVRNTQDALFKAIDRLLNYQKSVTSATADDSESQANHA

WP_175537819110_190 LNKAVDLGLANQNEQAREQLLGPVRPVQSAYFKALDDVIAYQKASTVSTADESERSAASA

WP_219952136110_207 VKKALEKGLANKNDEAREFILTDVRRAQDSLFKVIDTMLAYQKNATAVTADNSQSQAVNA

WP_272566864110_187 LNKAVDLGLANQNDQAREQLLGPVRPVQAAYFKALDDLVDYQKAATVRTADESEKAAV--

WP_284402436110_187 LNKAVDLGLANQNDQAREQLLGPVRPVQAAYFKALDDLVDYQKAATVRTADESEKAAV--

WP_033577122110_207 VRKAVEKGMENKDDEARNLLLTEVRRAQDGMFKAIDSMLAYQKNATAATADDSESQAENA

WP_226068163110_207 IKKAIEKGLANKDDEAREFIITDVRRAQDSLFKVIDTMLAYQKNATAVTADNSQSQAVNA

WP_022635338110_211 VGRAIEKGLENKDDDARNLILTDVRRAQDNMFKVIDSMLTYQKSATSTAADDSESQANSA

WP_253005091110_207 VKKAIEKGLANKDDEAREFIITEVRRAQDSLFKVIDTMLAYQKNATAVTADNSQSQAVNA

WP_198317810110_211 VRKAIDKGLENKDDEARDLILNDVRRSQDALFKAIDTMLTYQKSATTATADDSESQANGA

WP_284426831110_187 LNKAVDLGLANQNDQAREQLLGPVRPVQAAYFKALDDLVDYQKVATVRTADESEKDAV--

WP_239788193110_211 VRKTIELGLSNQPDNARNLILGDVRSTQDALFKVIENMLTYQKNATSTAADSSESQADNA

WP_175637768110_211 VGRAIEKGLENKDDDARNLILTDVRRAQENMFKVIDSLLAYQKSATSTTADDSESRASNA

WP_168641181112_190 LRRAIEKGMENKDDEARNLILTEVRRTQETLFKIIDTMLLYQKSATTTTANNSESQASNA

PacG MKKAIELAMTNQHEAFRNFLLTEVRAAQANVFTALDKMVERQKDLTVELANQSEKEALNA

WP_336700672110_207 VRKAVEKGMENKDDEARNLLLTEVRRAQDGMFKAIDSMLAYQKNATAATADDSESQAENA

WP_121574097110_189 VSKAISVGMSGQPEEARAVLFGELRDAQSGVFNALDAMISFQAKLTVDTANESQQQAISA

WP_317355727110_187 LNKAVDLGLANQNDQAREQLLGPVRPVQAAYFKALDDLVDYQKAATVRTADESEKDAV--

WP_316393554110_211 VRKTIELGLSNQSDKARNLILSDVRNAQEALFKVIETMLTYQKSATSTAADDSESQANSA

WP_407322000110_207 LKKALDKGLANKDDEARELIITDVRRAQDSLFKVIDTMLAYQKNATAVTADNSQSQAVNA

WP_374069696110_207 VGRAIEKGLENKDDDARNLILTDVRRAQDNMFKAIDSLLAYQKSATSTTADDSESRASNA

ATA2706911_184 VNKSIDTGMSGNIEETRGMLFGELRDAQNGIFDALDAMIDYQTTLTIETANASQHQATSA

WP_038917000110_207 IKKAIEKGLANKDDEAREFIITDVRRAQDSLFKVIDTMLAYQKNATAVTADNSQSQAVNA

WP_143839830110_207 VRKAVEKGMENKDDEARNLLLTEVRRAQDGMFKAIDSMLAYQKNATAATADDSESQAENA

WP_271465838110_207 LRKAEKLGVANDREGARSVILGELRTAQNAYFKAVDDMVDYQRKMTTALADASENDAASA

WP_038909929110_207 VGRAIEKGLENKDDDARNLILTDVRRAQENMFKVIDSLLAYQKSATSTTADDSESRASNA

WP_038909309110_207 LRKALEKGLANKDDEARELIITDVRRAQDSLFKIIDTMLAYQKNATTVTADNSQSQAVNA

WP_042867702111_190 LRRAIEKGMENKDDEARNLILTEVRRTQETLFKIIDTMLLYQKSATTTTANNSESQASNA

NPE69876110_207 VGRAIEKGLENKDDDARNLILTDVRRAQDNMFKAIDSLLAYQKSATSTTADDSESRASNA

WP_039693058110_211 VRKTIELGLSNQPDNARNLILSDVRNTQDALFKVIESMLTYQKNATSTAADSSESQADSA

WP_226099784110_207 LRKALEKGLENKDDEARELIITDVRRAQDSLFKIIDTMLAYQKNATTVTADNSQSQAVNA

190 200

| |

WP_284407211110_187 ----------------------

WP_248588623110_207 GVIMLSLAVLITLIGSL----I

WP_198317298110_211 GILMLSLAALIAVAGSLIAWLI

WP_137715051110_207 GSLMLISAVIAALLGSL----I

WP_161451117110_207 GILMLSLAALIAVTGSL----I

WP_209126666110_211 GILMLSLAALIAVAGSLIAWLI

WP_121480737110_211 GILMLSLAALIAVAGSLIAWLI

WP_198298549110_211 GILMLSLAALIAVAGSLIAWLI

MCL6405923110_207 GILMLSLAALIAVTGSL----I

WP_210182408110_207 GVIMLSLAVLITLVGSL----I

WP_038899989110_207 GILMLSLAALIAVTGSL----I

WP_198299881110_211 GILMLSLAALIAVAGSLIAWLI

WP_342699658110_207 GVIMLSLAVLITLIGSL----I

WP_264090912110_207 GTMMLIAVVIAALLGSL----I

WP_168361165110_207 GVIMLSLAVLITLVGSL----I

WP_226511591110_207 GVIMLSLAVLITLVGSL----I

WP_242751928110_211 GILMLSLAALIAVAGSLIAWLI

WP_013315853110_207 GILMLSLAALIAVTGSL----I

WP_192458598112_207 GLLMLSLAILIALAGSV----V

WP_198314307110_211 GILMLSLAALIAVAGSLIAWLI

WP_038921308110_211 GILMLSLAALIAVAGSLIAWLI

WP_406618536110_211 GILMLSLAALIAVAGSLIAWLI

WP_303313259110_211 GILMLSLAALIAVAGSLIAWLI

WP_038914843110_207 GVIMLSLAVLITLVGSL----I

WP_102802572110_207 GVIMLSLAVLITLVGSL----I

WP_220177006110_207 GVVMLSLAVLITLVGSL----I

WP_284431376110_187 ----------------------

WP_024104118110_211 GILMLSLAALIAVAGSLIAWLI

MCG8709529110_192 GRLM------------------

WP_336678565110_211 GILMLSLAALIAVAGSLIAWLI

WP_253940658110_211 GILMLSLAALIAVTGSLIAWLI

WP_171861319110_207 GILMLSLAALIAVTGSL----I

WP_284414662110_190 G---------------------

NMN9323418_205 GTMMLIAVVIAALLGSL----I

WP_284414646110_187 ----------------------

WP_198299430110_211 GILMLSLAALIAVAGSLIAWLI

WP_148042947110_207 GILMLSLAALIAVAGSL----V

RLM2168211_184 GSLMLSI---------------

WP_033568988110_207 GILMLSLAALIAVAGSL----V

WP_171850212110_207 GILMLSLAALIAVTGSL----I

WP_019845382110_207 GVIMLSLAVLITLVGSL----I

WP_272550727110_184 ----------------------

WP_146410942111_190 G---------------------

WP_17184960718_195 GILMLSLA--------------

WP_208228909110_195 GSLMLSI---------------

WP_113865726110_207 GTMMLIAVVIAALLGSL----I

WP_161132035110_211 GILMLSLAALIAVAGSLIAWLI

WP_201743379110_207 GILMLSLAALIAVAGSL----V

WP_038664436110_211 GILMLSLAALIAVAGSLIAWLI

WP_100850303110_211 GILMLSLAALIAVAGSLIAWLI

WP_012886618110_207 GVIMLSLAVLITLVGSL----I

WP_198314835110_211 GILMLSLAALIAVAGSLIAWLI

MDQ5892624110_207 GTLMVIIALLAVVLGAM----I

WP_038917453110_211 GILMLSLAALIAVAGSLIAWLI

WP_224062336110_207 GILMLSLAALIAVTGSL----I

WP_121589257110_195 GSLMLSI---------------

WP_048636656110_195 GSLMLSI---------------

WP_210173983110_207 GVIMLSLAVLIALVGSL----I

WP_253000914110_207 GVIMLSLAVLITLVGSL----I

WP_284603643110_207 GILMLSLAALIAVTGSL----I

WP_198298864110_211 GILMLSLAALIAVAGSLIAWLI

WP_172290490110_206 GSLMLIIAALVALLGGL----V

MEQ4512139110_207 GVIMLSLAVLITLVGSL----I

WP_023641026110_207 GVIMLSLAVLITLVGSL----I

WP_336684953110_207 GILMLSLAALIAVTGSL----I

WP_168366716110_207 GVIMLSLAVLITLVGSL----I

WP_150436034110_196 GILMLI---------------I

WP_198314030110_211 GILMLSLAALIAVAGSLIAWLI

WP_263064027110_211 GILMLSLAALIAVAGSLIAWLI

WP_049853775110_211 GILMLSLAALIAVAGSLIAWLI

WP_015855510112_207 GLLMLSLAILITLAGSV----I

WP_175537819110_190 G---------------------

WP_219952136110_207 GVIMLSLAVLITLVGSL----I

WP_272566864110_187 ----------------------

WP_284402436110_187 ----------------------

WP_033577122110_207 GILMLSLAVLIAVAGSL----V

WP_226068163110_207 GVIMLSLAVLITLVGSL----I

WP_022635338110_211 GILMLSLAALIAVAGSLIAWLI

WP_253005091110_207 GVVMLSLAVLITLVGSL----I

WP_198317810110_211 GILMLSLAALIAVAGSLIAWLI

WP_284426831110_187 ----------------------

WP_239788193110_211 GILMLSLAALIAVAGSLIAWLI

WP_175637768110_211 GILMLSLAALIAVTGSLIAWLI

WP_168641181112_190 G---------------------

PacG GTLMLIIALCASLLGGV----V

WP_336700672110_207 GILMLSLAVLIAVAGSL----V

WP_121574097110_189 G---------------------

WP_317355727110_187 ----------------------

WP_316393554110_211 GILMLSLAALIAVAGSLIAWLI

WP_407322000110_207 GVIMLSLAVLITLIGSL----I

WP_374069696110_207 GILMLSLAALIAVTGSL----I

ATA2706911_184 GSLMLSI---------------

WP_038917000110_207 GVIMLSLAVLITLVGSM----I

WP_143839830110_207 GILMLSLAVLIAVAGSL----V

WP_271465838110_207 GTAMVVLAVVSAALGAL----V

WP_038909929110_207 GILMLSLAALIAVTGSL----I

WP_038909309110_207 GVIMLSLAVLITLVGSL----I

WP_042867702111_190 G---------------------

NPE69876110_207 GILMLSLAALIAVTGSL----I

WP_039693058110_211 GILMLSLAALIAVAGSLIAWLI

WP_226099784110_207 GVIMLSLAVLIALVGSL----I
