# Supplementary material for: Chemotaxis to plant defense compounds in phytopathogens
Source: PLoS Pathog. 2026 May 20;22(5):e1014240. doi: 10.1371/journal.ppat.1014240 (PMC13215616; doi:10.1371/journal.ppat.1014240)
Supplement: S18 Fig — Pairwise sequence alignments of the LBDs of PacI and PacH with those of PcaY_PP (6) and PcpI (7). The alignment was done using the CLUSTALW algorithm of the NPS@ software (5). The Gonnet protein weight matrix was used; gap opening and gap extension penalties were 10.0 and 0.1, respectively. Residues in red are identical, green highly similar and blue weakly similar. (DOCX) [file ppat.1014240.s018.docx]

### **S18 Fig. Sequence alignment of four helix bundle LBDs of chemoreceptors that bind salicylate.** Pairwise sequence alignments of the LBDs of PacI and PacH with those of PcaY_PP (6) and PcpI (7). The alignment was done using the CLUSTALW algorithm of the NPS@ software (5). The Gonnet protein weight matrix was used; gap opening and gap extension penalties were 10.0 and 0.1, respectively. Residues in red are identical, green highly similar and blue weakly similar.

**PacI-PcaY**

10 20 30 40 50 60

| | | | | |

PacI_LBD R--THLVGLGNTTDNLAKNHLANLIVLQELKDNLNVTIKATLRMLITTEKKVLEDNQKLI

PcaY_LBD GSDQQITELDQTAHQ--SDRLN-NALLMAIRSSANVSSG-FIEQLGGHDESAGKRMALSV

70 80 90 100 110 120

| | | | | |

PacI_LBD ETTSARNAKLVTQLEENLQAKEVRNILGELQQNRTEFATV-GRQSVALSLNNKQAESIEL

PcaY_LBD E-LNNKSQALVDEFVENAREPALRGLATELQATFAEYAKAVAGQREA-----TRQRSLE-

130 140 150 160

| | | |

PacI_LBD VRTQLEPIQTKLFN---NLNTMIQLQKDYTTQTATNAIEESYYDGNSL

PcaY_LBD ---QYFKVNSDAGNAMGRLQTLRQQLVTTLSERGQQIMLESDRRLARA

**PacH-PcaY**

10 20 30 40 50 60

| | | | | |

PacH_LBD RVHLLDLSEDIESLSEKNLTSLILIQDAKSGFDAVARSVRTIGLTSDSSRIQEEKRLIDQ

PcaY_LBD ---------------------------------------------GSDQQITELDQTAHQ

70 80 90 100 110 120

| | | | | |

PacH_LBD QIALNTDILTKLYSHLSE----PESRDSLDRLTQARPAYRDAVNKAVELGVSENAE-ERA

PcaY_LBD SDRLNNALLMAIRSSANVSSGFIEQLGGHDESAGKRMALSVELNNKSQALVDEFVENARE

130 140 150 160 170 180

| | | | | |

PacH_LBD RAVQLMVNEMQITQAPVFAALDSMTELQKKRTMEMTTSAMQEARSDGN------------

PcaY_LBD PALRGLATELQATFAEYAKAVAGQREATRQRSLEQYFKVNSDAGNAMGRLQTLRQQLVTT

190

|

PacH_LBD ----------------TLI

PcaY_LBD LSERGQQIMLESDRRLARA

**PacI-PcpI**

10 20 30 40 50 60

| | | | | |

PacI_LBD RTHLVGLGNTTDNLAKNHLANLIVLQELKDNLNVTIKA-----------TLRMLITTEKK

PcpI_LBD ATGWRGMDGIIERG--DKLGNISVIHQRTLELRIARQAYVIKPDSTTTAQIETALDNLEQ

70 80 90 100 110 120

| | | | | |

PacI_LBD VLEDNQKLIETTSARNAKLVTQLEENLQAKEVRNILGELQQNRTEFATVGRQSVALSLNN

PcpI_LBD QIQRMQPLIEKPSDQ-QRLAQQLDA---ARQYRQLFADYRQ-----------AAAGSTAA

130 140 150 160

| | | |

PacI_LBD KQAES--IELVRTQ------LEPIQTKLFNNLNTMIQLQKDYTTQTAT

PcpI_LBD SQALQRMADLGGRLLETSQAMTVSQTKVRNA----DARQAKTLLGGAT

**PacH-PcpI**

10 20 30 40 50 60

| | | | | |

PacH_LBD RVHLLDLSEDIESLSEKNLTSLILIQDAKSGFDAVARSVRTIGLTS-DSSRIQEEKRLID

PcpI_LBD ATGWRGMDGIIERGDKLG--NISVIHQ-RTLELRIARQAYVIKPDSTTTAQIETALDNLE

70 80 90 100 110 120

| | | | | |

PacH_LBD QQIALNTDILTKLYSHLSEPESRDSLDRLTQARPAYRDAVNKAVELGVSENAEERARAVQ

PcpI_LBD QQIQRMQPLIEKPSDQQRLAQQLDAARQYRQLFADYRQAAA-----GSTAASQALQRMAD

130 140 150 160

| | | |

PacH_LBD LMVNEMQITQAPVFAALDSMTELQKKRTMEMTTSAMQEARSDGNTLI

PcpI_LBD LGGRLLETSQA--------MTVSQTK----VRNADARQAKTLLGGAT
