# Supplementary material for: Chemotaxis to plant defense compounds in phytopathogens
Source: PLoS Pathog. 2026 May 20;22(5):e1014240. doi: 10.1371/journal.ppat.1014240 (PMC13215616; doi:10.1371/journal.ppat.1014240)
Supplement: S1 Table — Data were extracted from (8). The authors clustered all available chemoreceptor LBD sequences. They then quantified the relative amount of chemoreceptors from plant-associated bacteria (PAB) and plant pathogens (PP) in each of the LBD clusters. The DPS score corresponds to the abundance of receptors from PAB and PP in each of the clusters. A score of 100 indicates that all sequences of a cluster are from PAB/PP and a score of 0 indicates none. Names and ligands of characterized chemoreceptors are also indicated. The chemoreceptors studied in this article are in bold. (DOCX) [file ppat.1014240.s019.docx]

**S1 Table. Degree of plant specificity (DPS) scores of the** ***P. atrosepticum* SCRI1043 chemoreceptors**. Data were extracted from (8). The authors clustered all available chemoreceptor LBD sequences. They then quantified the relative amount of chemoreceptors from plant-associated bacteria (PAB) and plant pathogens (PP) in each of the LBD clusters. The DPS score corresponds to the abundance of receptors from PAB and PP in each of the clusters. A score of 100 indicates that all sequences of a cluster are from PAB/PP and a score of 0 indicates none. Names and ligands of characterized chemoreceptors are also indicated. The chemoreceptors studied in this article are in bold.

| **Locus tag** | **Name** | **Ligand** | **Cluster number** | **Sequences per cluster** | **DPS score** | | **Ref.** |
| --- | --- | --- | --- | --- | --- | --- | --- |
|  |  |  |  |  | **PAB** | **PP** |  |
| ECA_RS00400 |  |  | 838 | 17 | 80 | 80 |  |
| ECA_RS00455 |  |  | 409 | 35 | 63.64 | 48.48 |  |
| ECA_RS00895 |  |  | 159 | 82 | 43.66 | 16.9 |  |
| ECA_RS00900 |  |  | 159 | 82 | 43.66 | 16.9 |  |
| ECA_RS02005 |  |  | 42 | 213 | 51.09 | 14.6 |  |
| ECA_RS02210 | PacN | Nitrate | 50 | 189 | 10 | 8.24 | (2) |
| ECA_RS02220 |  |  | 593 | 25 | 80 | 20 |  |
| ECA_RS05475 | PacB | Amino acids | 158 | 82 | 30.77 | 12.82 | (9) |
| ECA_RS06345 |  |  | 36 | 226 | 32.82 | 12.21 |  |
| ECA_RS06625 |  |  | 123 | 102 | 61.22 | 42.86 |  |
| ECA_RS07510 |  |  | 116 | 106 | 56.99 | 29.01 |  |
| ECA_RS08330 |  |  | 179 | 77 | 68.09 | 40.43 |  |
| ECA_RS08370 | PacC | Amino acids | 20 | 279 | 48.45 | 13.92 | (9) |
| ECA_RS08780 | Not listed | | | | | | |
| ECA_RS09870 |  |  | 2 | 3814 | 31.09 | 3.51 |  |
| ECA_RS09875 |  |  | 2 | 3814 | 31.09 | 3.51 |  |
| ECA_RS10160 |  |  | 517 | 29 | 34.48 | 17.24 |  |
| ECA_RS10935 | PacA | Quaternary amines/proline | 96 | 123 | 34.48 | 8.62 | (10) |
| ECA_RS11380 |  |  | 269 | 53 | 54 | 32 |  |
| ECA_RS12390 | PacP | Phosphorylated C3 compounds | 160 | 81 | 38.46 | 12.82 | (3) |
| ECA_RS12635 |  |  | 485 | 32 | 75.86 | 41.38 |  |
| ECA_RS12640 |  |  | 1260 | 10 | 90 | 60 |  |
| ECA_RS13300 |  |  | 123 | 102 | 61.22 | 42.86 |  |
| ECA_RS15955 |  |  | 1417 | 8 | 75 | 75 |  |
| ECA_RS17685 |  |  | 2 | 1140 | 22.68 | 4.84 |  |
| ECA_RS17750 |  |  | 932 | 15 | 100 | 100 |  |
| ECA_RS17860 | PacF | Formate | 15 | 307 | 32.97 | 8.7 | (1) |
| ECA_RS17910 |  |  | 533 | 29 | 79.31 | 65.52 |  |
| ECA_RS18000 |  |  | 18 | 287 | 39.86 | 6.99 |  |
| ECA_RS18955 |  |  | 179 | 77 | 68.09 | 40.43 |  |
| ECA_RS19280 |  |  | 179 | 77 | 68.09 | 40.43 |  |
| ECA_RS20365 |  |  | 630 | 24 | 100 | 87.5 |  |
| **ECA_RS21440** | **PacH** |  | **246** | **58** | **54.9** | **21.57** |  |
| **ECA_RS21445** | **PacI** |  | **630** | **24** | **100** | **87.5** |  |
| **ECA_RS21450** |  |  | **883** | **16** | **85.71** | **78.57** |  |
| **ECA_RS21455** | **PacG** |  | **883** | **16** | **85.71** | **78.57** |  |
