# Supplementary material for: Chemotaxis to plant defense compounds in phytopathogens
Source: PLoS Pathog. 2026 May 20;22(5):e1014240. doi: 10.1371/journal.ppat.1014240 (PMC13215616; doi:10.1371/journal.ppat.1014240)
Supplement: S2 Table — It is indicated whether the corresponding strain is a plant-associated bacterium (PAB) or a phytopathogen (PP). Data are taken from (8). (DOCX) [file ppat.1014240.s020.docx]

**S2 Table. Composition of the LBD clusters that contain chemoreceptors PacH, PacI and PacG**. It is indicated whether the corresponding strain is a plant-associated bacterium (PAB) or a phytopathogen (PP). Data are taken from (8).

| **Cluster 246 (PacH)** | | | |
| --- | --- | --- | --- |
| **Locus tag** | **strain** | **PAB** | **PP** |
| bpln_2g15840 | [*Burkholderia plantarii* strain ATCC 43733](https://www.ncbi.nlm.nih.gov/nuccore/CP007213.1) | X | X |
| bpln_2g27810 | [*Burkholderia plantarii* strain ATCC 43733](https://www.ncbi.nlm.nih.gov/nuccore/CP007213.1) | X | X |
| Anae109_3837 | *Anaeromyxobacter* sp. Fw109-5 | - | - |
| SAMN05216359_11964 | *Roseateles* sp. YR242 | - | - |
| GCA_001598255_02006 | *Mitsuaria chitosanitabida* ATCC BAA-476 | - | - |
| GCA_001598255_02258 | *Mitsuaria chitosanitabida* ATCC BAA-476 | - | - |
| SAMN05428960_2928 | *Roseateles* sp. YR242 | - | - |
| E1O_25750 | *Burkholderiales* bacterium GJ-E10 | - | - |
| WB66_05005 | Bacteria symbiont BFo1 of *Frankliniella occidentalis* strain SwAb130 | - | - |
| Hrubri_2649 | *Herbaspirillum rubrisubalbicans* M1 | X | - |
| B5S52_00615 | *Pectobacterium brasiliense strain* SX309 | X | X |
| OI69_10990 | *Pectobacterium fontis* strain M022 | X | X |
| IP80_13030 | Beta proteobacterium AAP65 | - | - |
| IP80_20755 | Beta proteobacterium AAP65 | - | - |
| ASR47_1005182 | *Janthinobacterium psychrotolerans* strain S3-2 | - | - |
| PMI16_02566 | *Herbaspirillum* sp. CF444 | X | - |
| PMI16_04933 | *Herbaspirillum* sp. CF444 | X | - |
| GCA_001189965_01861 | *Herbaspirillum rhizosphaerae* | X | - |
| F506_09990 | *Herbaspirillum hiltneri* N3 | X | - |
| GCA_000577615_00122 | *Herbaspirillum* sp. RV1423 | X | - |
| GCA_000577615_00481 | *Herbaspirillum* sp. RV1423 | X | - |
| GCA_000383275_01634 | *Paraburkholderia caledonica* | X |  |
| AC233_27740 | *Burkholderia* sp. HB1 | - | - |
| BC1003_5051 | *Burkholderia* sp. CCGE1003 | X |  |
| BUPH_00933 | [*Paraburkholderia phenoliruptrix* BR3459a](https://www.ncbi.nlm.nih.gov/taxonomy/1229205) | X |  |
| SAMN05192548_100610 | *Roseateles* sp. YR242 | - | - |
| GCA_000472445_00164 | *Burkholderia* sp. WSM2230 | X |  |
| BN1221_01353c | [*Brenneria goodwinii* strain OBR1](https://www.ncbi.nlm.nih.gov/nuccore/CGIG00000000.1) | X | X |
| PC1_0111 | *Pectobacterium carotovorum* subsp. carotovorum PC1 | X | X |
| PC1_0113 | *Pectobacterium carotovorum* subsp. carotovorum PC1 | X | X |
| EV46_21570 | *Pectobacterium atrosepticum* strain JG10-08 | X | X |
| BG57_23745 | *Caballeronia grimmiae* strain R27 | - | - |
| NG42_01585 | *Erwinia iniecta* strain B120 | - | - |
| GCA_000336255_04440 | *Erwinia toletana* DAPP-PG 735 | X |  |
| BM43_4417 | *Burkholderia gladioli* strain ATCC 10248 | X | X |
| PI87_25685 | *Ralstonia* sp. A12 | - | - |
| KP22_20145 | *Pectobacterium betavasculorum* strain NCPPB 2795 | X | X |
| KP22_20150 | *Pectobacterium betavasculorum* strain NCPPB 2795 | X | X |
| SAMN02982919_01219 | *Roseateles* sp. YR242 | - | - |
| GCA_000341045_01959 | *Paraburkholderia kururiensis* M130 | X | - |
| ASC93_03965 | *Massilia* sp. Root335 | X | - |
| SAMN05428966_106328 | *Roseateles* sp. YR242 | - | - |
| ASD28_30425 | *Massilia* sp. Root335 | X | - |
| SAMN05445871_6163 | *Roseateles* sp. YR242 | - | - |
| GCA_001571305_02128 | *Erwinia persicina* NBRC 102418 | X | X |
| GWL_20470 | *Herbaspirillum* sp. GW103 | X |  |
| AWB75_05472 | *Burkholderia catudaia* type strain LMG 29318 | - | - |
| AWB73_06831 | *Caballeronia turbans* isolate LMG 29316 | - | - |
| HFRIS_013499 | *Herbaspirillum frisingense* GSF30 | X |  |
| GCA_000745265_04419 | *Massilia* sp. 9096 | - | - |
| SAMN05192589_11765 | *Roseateles* sp. YR242 | - | - |
| WM40_11660 | *Robbsia andropogonis* strain ICMP2807 | X | - |
| SAMN04489710_11616 | *Roseateles* sp. YR242 | - | - |
| GCA_001941825_04103 | *Massilia putida* | - | - |
| GCA_001307855_01072 | *Rhodocyclaceae bacterium* Paddy-1 | - | - |
| Dsui_1436 | *Azospira oryzae* PS | - | - |
| RSp1363 | *Ralstonia solanacearum* GMI1000 | X | X |
| GCA_001282395_01169 | *Paracidovorax avenae* | X | X |
| **Cluster 630 (PacI)** | | | |
| B5S52_00620 | *Pectobacterium brasiliense* strain SX309 | X | X |
| B5S52_00625 | *Pectobacterium brasiliense* strain SX309 | X | X |
| B5S52_01635 | *Pectobacterium brasiliense* strain SX309 | X | X |
| OI69_05285 | *Pectobacterium fontis* strain M022 | X | X |
| LH89_13380 | *Dickeya fangzhongdai* strain ND14b | X | X |
| LH89_13385 | *Dickeya fangzhongdai* strain ND14b | X | X |
| GCA_000406285_03679 | *Dickeya* sp. DW 0440 | X | X |
| GCA_000406285_03680 | *Dickeya* sp. DW 0440 | X | X |
| PC1_0112 | *Pectobacterium carotovorum* subsp. carotovorum PC1 | X | X |
| PC1_3910 | *Pectobacterium carotovorum* subsp. carotovorum PC1 | X | X |
| EV46_20505 | *Pectobacterium atrosepticum* strain JG10-08 | X | X |
| EV46_21580 | *Pectobacterium atrosepticum* strain JG10-08 | X | X |
| EV46_21575 | *Pectobacterium atrosepticum* strain JG10-08 | X | X |
| Dda3937_02028 | *Dickeya dadantii* 3937 | X | X |
| Dd1591_4046 | *Dickeya chrysanthemi* Ech1591 | X | X |
| GCA_000365405_00116 | [Dickeya dianthicola NCPPB 3534](https://www.ncbi.nlm.nih.gov/data-hub/genome/GCF_000365405.2/) | X | X |
| W5S_0107 | *Pectobacterium parmentieri* strain SCC3193 | X | X |
| W5S_4243 | *Pectobacterium parmentieri* strain SCC3193 | X | X |
| KP22_13635 | *Pectobacterium betavasculorum* NCPPB 2795 | X | X |
| Dd586_4022 | *Dickeya parazeae* Ech586 | X | X |
| Ser39006_00334 | Serratia sp. strain ATCC 39006 | X | - |
| GCA_001969245_04188 | *Delftia acidovorans* | X | - |
| Dd703_3862 | *Dickeya dadantii* Ech703 | X | X |
| Dd703_3864 | *Dickeya dadantii* Ech703 | X | X |
| Dd703_3864 | *Dickeya dadantii* Ech703 | X | X |
| GCA_000406125_00127 | *Dickeya poace iphila* | X | X |
| **Cluster 883 (PacG and ECA_RS21450)** | | | |
| AB182_18700 | *Phytobacter ursingii* strain CAV1151 | X | - |
| B5S52_00610 | *Pectobacterium brasiliense* strain SX309 | X | X |
| OI69_15610 | *Pectobacterium fontis* strain M022 | X | X |
| LH89_13370 | *Dickeya fangzhongdai* strain ND14b | X | X |
| BrE312_4275 | *Brenneria* sp. EniD312 | X | - |
| EV46_21580 | *Pectobacterium atrosepticum* strain JG10-08 | X | X |
| EV46_21585 | *Pectobacterium atrosepticum* strain JG10-08 | X | X |
| Dda3937_02025 | *Dickeya dadantii* 3937 | X | X |
| Dd1591_4048 | *Dickeya chrysanthemi* Ech1591 | X | X |
| GCA_000365405_00113 | *Dickeya dianthicola* NCPPB 3534 | X | X |
| W5S_0105 | *Pectobacterium parmentieri* strain SCC3193 | X | X |
| W5S_0106 | *Pectobacterium parmentieri* strain SCC3193 | X | X |
| Dd586_4025 | *Dickeya parazeae* Ech586 | X | X |
| A3780_05710 | *Kosakonia radicincitans* strain GXGL-4A | - | - |
| AKI40_3735 | *Enterobacter* sp. FY-07 | - | - |
| GCA_000406125_00123 | *Dickeya poaceiphila* | X | X |
