# Supplementary material for: Chemotaxis to plant defense compounds in phytopathogens
Source: PLoS Pathog. 2026 May 20;22(5):e1014240. doi: 10.1371/journal.ppat.1014240 (PMC13215616; doi:10.1371/journal.ppat.1014240)
Supplement: S3 Table — A) Sequence identities of 4 chemoreceptors from the gene cluster of P. atrosepticum SCRI1043 studied in this work. B) Sequence identities between the four-helix bundle type LBDs of chemoreceptors that bind salicylate. The LBDs of the PcaY-PP and PcpI chemoreceptors from Pseudomonas putida KT2440 and P. putida 1290 bound salicylate (6, 7). Alignments were made with the BLAST tool of NCBI. The LBD was defined as the protein segment between the two transmembrane regions as predicted by TMHMM – 2.0 (11). (DOCX) [file ppat.1014240.s021.docx]

### **S3 Table. Amino acid sequence identity (%) of pairwise alignments of the LBDs from different chemoreceptors**. **A)** Sequence identities of 4 chemoreceptors from the gene cluster of *P. atrosepticum* SCRI1043 studied in this work. **B)** Sequence identities between the four helix bundle type LBDs of chemoreceptors that bind salicylate. The LBDs of the PcaY-PP and PcpI chemoreceptors from *Pseudomonas putida* KT2440 and *P. putida* 1290 bound salicylate (6, 7). Alignments were made with the BLAST tool of NCBI. The LBD was defined as the protein segment between the two transmembrane regions as predicted by TMHMM – 2.0 (11).

**A**

| LBD | ECA_RS21440  (PacH) | ECA_RS21445  (PacI) | ECA_RS21450 | ECA_RS21455 (PacG) |
| --- | --- | --- | --- | --- |
| ECA_RS21440 (PacH) | X | 26 | 29 | 34 |
| ECA_RS21445(PacI) |  | X | 25 | 26 |
| ECA_RS21450 |  |  | X | 51 |
| ECA_RS21455 (PacG) |  |  |  | X |

**B**

| LBD | PacI | PacH | PcaY_PP | PcpI |
| --- | --- | --- | --- | --- |
| PacI | x | 26 | 17 | 18 |
| PacH |  | x | 13 | 16 |
| PcaY_PP |  |  | x | 19 |
| PcpI |  |  |  | x |
