# Supplementary material for: Chemotaxis to plant defense compounds in phytopathogens
Source: PLoS Pathog. 2026 May 20;22(5):e1014240. doi: 10.1371/journal.ppat.1014240 (PMC13215616; doi:10.1371/journal.ppat.1014240)
Supplement: S4 Table — Microcalorimetric titrations were conducted with the highest possible ligand concentrations, i.e., that induced dilution heats inferior to 0.1 μcal/sec when injected into buffer. These concentrations were in the range of 1–5 mM. Listed are the compounds that did not produce binding heats when injected into 32–100 μM protein solutions. (DOCX) [file ppat.1014240.s022.docx]

**S4 Table. List of compounds that failed to bind in microcalorimetric experiments to the LBDs of chemoreceptors PacH, PacI and PacG.** Microcalorimetric titrations were conducted with the highest possible ligand concentrations, i.e. that induced dilution heats inferior to 0.1 μcal/sec when injected into buffer. These concentrations were in the range of 1 to 5 mM. Listed are the compounds that did not produce binding heats when injected into 32 to 100 μM protein solutions.

| LBD | Ligands that failed to bind |
| --- | --- |
| PacH  (ECA_RS21440) | benzoate, vanillate, *m*-hydroxybenzoate, *p*-hydroxybenzoate, *p*-hydroxybenzaldehyde, *o*-aminobenzoate, *o*-chlorobenzoate, *o*-nitrobenzoate, *o*-methoxybenzoate, *m*-aminobenzoate, *m*-chlorobenzoate, *m*-nitrobenzoate, *m*-methoxybenzoate, *p*-aminobenzoate, *p*-chlorobenzoate, *p*-nitrobenzoate, *p*-methoxybenzoate, protocatechuate, quinate, shikimate, adipate, sorbate, agmatine |
| PacI  (ECA_RS21445) | vanillate, vanilline, *p*-nitrobenzoate, protocatechuate, *o*-aminobenzoate, *o*-chlorobenzoate, *o*-methoxybenzoate, *o*-nitrobenzoate, *m*-aminobenzoate, *m*-chlorobenzoate, *m*-hydroxybenzoate, *m*-methoxybenzoate, *m*-nitrobenzoate, *p*-aminobenzoate, *p*-hydroxybenzaldehyde, quinate, shikimate, adipate, formate, caprate, agmatine |
| PacG  (ECA_RS21455) | *o*-hydroxybenzoate, vanillin, *p*-coumarate, ferulate |
