# Supplementary material for: Chemotaxis to plant defense compounds in phytopathogens
Source: PLoS Pathog. 2026 May 20;22(5):e1014240. doi: 10.1371/journal.ppat.1014240 (PMC13215616; doi:10.1371/journal.ppat.1014240)
Supplement: S5 Table — Statistics for the highest-resolution shell are shown in parentheses. (DOCX) [file ppat.1014240.s023.docx]

**S5 Table. Resolution of the three-dimensional structure of PacG-LBD by X-ray crystallography: Data collection and refinement statistics.** Statistics for the highest-resolution shell are shown in parentheses.

| **Data collection** |  |  |
| --- | --- | --- |
| Synchrotron / beamline | XALOC / ALBA | ID23-2 / ESRF |
| Space group | *P 2_1_ 2_1_ 2* | *P 1 2_1_ 1* |
| Cell dimensions |  |  |
| *a*, *b*, *c* (Å) | 29.62 56.50 83.28 | 29.49 57.53 84.00 |
| α, β, γ (°) | 90.00, 90.00, 90.00 | 90.00, 90.99, 90.00 |
| Resolution range | 46.75 - 1.4 (1.42 - 1.4) | 83.99 - 1.8 (1.84 - 1.8) |
| Completeness (%) | 99.7 (100.0) | 98.1 (97.9) |
| Multiplicity | 4.6 (4.7) | 3.5 (3.5) |
| Mean I/sigma (I) | 7.6 (0.7) | 5.9 (0.9) |
| Wilson B-factor (Å^2^) | 21.53 | 15.3 |
| R-merge | 0.064 (1.609) | 0.122(1.33) |
| CC1/2 | 0.998 (0.553) | 0.993 (0.478) |
| **Refinement** |  |  |
| No. reflections | 28,155 (2,762) | 25,581 (2,803) |
| *R*_work_ / *R*_free_ | 19.65/21.80 | 17.93/22.63 |
| No. atoms | 1313 | 2785 |
| Protein | 1227 | 2521 |
| Ligand/ion | 28 | 45 |
| Water | 58 | 219 |
| R.m.s. deviations |  |  |
| Bond lengths (Å) | 0.006 | 0.014 |
| Bond angles (°) | 0.84 | 1.24 |
| Ramachandran |  |  |
| favored (%) | 97.90 | 99.30 |
| outliers (%) | 0.00 | 0.00 |
| Average B-factor (Å^2^) | 38.91 | 30.43 |
| macromolecules | 38.92 | 30.31 |
| ligands | 40.22 | 29.57 |
| solvent | 37.92 | 32.08 |
| PDB ID | 9Q8B | 9Q8E |
