# Supplementary material for: Chemotaxis to plant defense compounds in phytopathogens
Source: PLoS Pathog. 2026 May 20;22(5):e1014240. doi: 10.1371/journal.ppat.1014240 (PMC13215616; doi:10.1371/journal.ppat.1014240)
Supplement: S6 Table — (DOCX) [file ppat.1014240.s024.docx]

**S6 Table. Closest structural homologs of PacG-LBD as derived from a structural alignment using DALI** (12) **with all 3D structures deposited in the protein data bank.**

| **Name** | **Protein family** | **Species** | **Domain family,**  **Pfam** | **Ligands** | **Pdb ID** | **Z** | **rmsd** | **lali** | **nres** | **%id** | **Ref.** |
| --- | --- | --- | --- | --- | --- | --- | --- | --- | --- | --- | --- |
| PcaY_PP | Chemoreceptor | *Pseudomonas aeruginosa* | TarH  PF02203 | Benzoate derivatives | 6s18 | 18.7 | 2.0 | 140 | 143 | 7 | (13) |
| MCP2201 | Chemoreceptor | *Comamonas testosteroni* | TarH  PF02203 | Citrate | 6its | 18.2 | 2.2 | 143 | 147 | 16 | (14) |
| HK9 | Histidine sensor kinase | *Rhodopseudomonas palustris* | CHASE 3  PF05227 | - | 3va9 | 16.7 | 1.8 | 123 | 127 | 13 | Unpublished |
| Adeh_2942 | Histidine sensor kinase | *Anaeromyxobacter dehalogenans* | Un-annotated | - | 4k0d | 15.7 | 2.4 | 140 | 145 | 9 | (15) |
| - | Hbc599-membrane-protein-binder | artificial protein | Cytochrome B562  PF07361 |  | 9ivk | 14.5 | 2.5 | 135 | 278 | 14 | (16) |
| - | Hbc599-in-complex-with-wfap1.1 | artificial protein | Un-annotated | WFAP1.1 | 8w6e | 14.5 | 2.6 | 134 | 161 | 15 | (16) |
| McpN | Chemoreceptor | *Pseudomonas aeruginosa* | PilJ  PF13675 | Nitrate | 6gcv | 13.8 | 2.4 | 118 | 126 | 12 | (17) |
| VC1313 | Chemoreceptor | *Vibrio cholerae* | 4HB_MCP_1  PF12729 | D-lysine | 8bsb | 13.3 | 2.6 | 136 | 162 | 10 | (18) |
