# Supplementary material for: Chemotaxis to plant defense compounds in phytopathogens
Source: PLoS Pathog. 2026 May 20;22(5):e1014240. doi: 10.1371/journal.ppat.1014240 (PMC13215616; doi:10.1371/journal.ppat.1014240)
Supplement: S7 Table — (DOCX) [file ppat.1014240.s025.docx]

**S7 Table. Strains and plasmids used in this study**.

| **Strains and plasmids** | **Genotype or relevant characteristics** | **Ref.** |
| --- | --- | --- |
| **Strains** | | |
| *Escherichia coli* BL21(DE3) | F^–^ *ompT* *gal* *dcm* *lon* *hsdS_B_*(*r_B_*^–^*m_B_*^–^) λ(DE3 [*lacI* *lacUV5*-*T7p07* *ind1* *sam7* *nin5*]) [*malB*^+^]_K-12_(λ^S^) | (19) |
| *E. coli* DH5α | F^–^ *endA1* *glnV44* *thi-1*  *recA1*  *relA1*  *gyrA96 deoR* *nupG* *purB20* φ80d*lacZ*ΔM15 Δ(*lacZYA-argF*)U169, hsdR17(*r_K_*^–^*m_K_*^+^), λ^–^ | (20) |
| *E. coli* CC118λ*pir* | *araD* Δ(*ara*, *leu*) Δ*lacZ74 phoA20 galK thi-1 rspE rpoB argE recA1* λ*pir* | (21) |
| *E. coli* β2163 | F- RP4-2-Tc::Mu Δ*dapA*::(*erm-pir*); Km^R^, Em^R^ | (22) |
| *Pectobacterium atrosepticum* SCRI1043 | Wild type strain | (23) |
| *P. atrosepticum* SCRI1043 Δ*pacH* | SCRI1043 deletion mutant of *ECA_RS21440* (*pacH*) | This study |
| *P. atrosepticum* SCRI1043 Δ*pacI* | SCRI1043 deletion mutant of *ECA_RS21445* (*pacI*) | This study |
| *P. atrosepticum* SCRI1043 Δ*pacG* | SCRI1043 deletion mutant of *ECA_RS21455* (*pacG*) | This study |
| PacHIG | SCRI1043 Δ*pacH-pacI-ECA_RS24450-pacG* | This study |
| *P. atrosepticum* SCRI1043 Δ*cheA* | SCRI1043 deletion mutant of *ECA_RS08360* (*cheA*) | (24) |
| *P. atrosepticum* SCRI1043 Δ*pacC* | SCRI1043 deletion mutant of *ECA_RS08370* (*pacC*) | (9) |
| *P. atrosepticum* SCRI1043 Δ*pacF* | SCRI1043 deletion mutant of *ECA_RS17860* (*pacF*) | (1) |
| *P. atrosepticum* SCRI1043 Δ*pacN* | SCRI1043 deletion mutant of *ECA_RS02210* (*pacN*) | (2) |
| *P. atrosepticum* SCRI1043 Δ*pacP* | SCRI1043 deletion mutant of *ECA_* *RS12390* (*pacP*) | (3) |
| **Plasmids** | | |
| pET28b(+) | Km^R^; protein expression plasmid | Novagen |
| pET28b-ECA_RS21440-LBD | Km^R^; pET28b(+) derivative encoding ECA_RS21440 (PacH) LBD | GenScript |
| pET28b-ECA_RS21445-LBD | Km^R^; pET28b(+) derivative encoding ECA_RS21445 (PacI) LBD | GenScript |
| pET28b-ECA_RS21450-LBD | Km^R^; pET28b(+) derivative encoding ECA_RS21450 LBD | GenScript |
| pET28b-ECA_RS21455-LBD | Km^R^; pET28b(+) derivative encoding ECA_RS21455 (PacG*)* LBD | GenScript |
| pUC18Not | Ap^R^; identical to pUC18 but with two NotI sites flanking pUC18 polylinker | (21) |
| pUC18_ΔECA_RS21440 | Ap^R^; 1.5-kb PCR product containing a 1,463 bp deletion of *ECA_ RS21440* (*pacH*) inserted into the EcoRI/PstI sites of pUC18Not | This study |
| pUC18_ΔECA_RS21440-km3 | Ap^R^, Km^R^; 950 bp BamHI fragment containing km3 cassette of p34S-Km3 was inserted into the same site of pUC18_ΔECA_ RS21440 | This study |
| pUC18_ΔECA_RS21445 | Ap^R^; 1.5-kb PCR product containing a 1,310 bp deletion of *ECA_ RS21445* (*pacI*) inserted into the EcoRI/PstI sites of pUC18Not | This study |
| pUC18_ΔECA_RS21445-km3 | Ap^R^, Km^R^; 950 bp BamHI fragment containing km3 cassette of p34S-Km3 was inserted into the same site of pUC18_ΔECA_ RS21445 | This study |
| pUC18_ΔECA_RS21455 | Ap^R^; 1.5-kb PCR product containing a 1,468 bp of the *ECA_ RS21455* (*pacG*) up- and downstream sequences inserted into the BamHI/HindIII sites of pUC18Not | This study |
| pUC18_ΔECA_RS21455-km3 | Ap^R^, Km^R^; 950 bp PstI fragment containing km3 cassette of p34S-Km3 was inserted into the same site of pUC18_ΔECA_ RS21455 | This study |
| pUC18_PacHIG | Ap^R^; 1.5-kb PCR product containing 700 bp of *ECA_ RS21440* and 692 bp of *ECA_ RS21455* inserted into the SphI/BamHI sites of pUC18Not | This study |
| pUC18_PacHIG-km3 | Ap^R^, Km^R^; 0.95-kb PstI fragment containing km3 cassette of p34S-Km3 was inserted into the same site of pUC18_ PacHIG | This study |
| pKNG101 | Sm^R^; *oriR6K mob sacBR* | (25) |
| pKNG101_ΔECA_RS21440 | Sm^R^, Km^R^; 2.6-kb NotI fragment of pUC18NotI-ECA_RS21440-Km3 was cloned at the same site in pKNG101 | This study |
| pKNG101_ΔECA_RS21445 | Sm^R^, Km^R^; 2.6-kb NotI fragment of pUC18NotI-ECA_RS21445-Km3 was cloned at the same site in pKNG101 | This study |
| pKNG101_ΔECA_RS21455 | Sm^R^, Km^R^; 2.6-kb NotI fragment of pUC18NotI-ECA_RS21455-Km3 was cloned at the same site in pKNG101 | This study |
| pKNG101_PacHIG | Sm^R^, Km^R^; 2.6-kb NotI fragment of pUC18_PacHIG-Km3 was cloned at the same site in pKNG101 | This study |
| pBBR1MCS-5_START (pBBR empty) | Gm^R^; *oriRK2 mobRK2* | (26) |
| pBBR*cheA* | Gm^R^; a 2.1-kb PCR fragment containing the *cheA* (*ECA_RS08360*) gene cloned into the NdeI/PstI sites of pBBR1MCS-5_START | This study |
| pBBR*pacG* | Gm^R^; a 1.7-kb PCR fragment containing the *pacG* (*ECA_RS21455*) gene cloned into the NdeI/EcoRI sites of pBBR1MCS-5_START | This study |

*^a^*Em, erythromycin; Ap, ampicillin; Km, kanamycin; Sm, streptomycin; Gm, gentamicin
