# Supplementary material for: Chemotaxis to plant defense compounds in phytopathogens
Source: PLoS Pathog. 2026 May 20;22(5):e1014240. doi: 10.1371/journal.ppat.1014240 (PMC13215616; doi:10.1371/journal.ppat.1014240)
Supplement: S8 Table — (DOCX) [file ppat.1014240.s026.docx]

**S8 Table.** **Oligonucleotides used in this study.**

| **Name** | **Sequence (5’-3’)** | **Purpose** |
| --- | --- | --- |
| RS21440-EcoRI-F | TAATGAATTCGGCAAAGTGCTGGTGGAG | Construction of pUC18_ΔECA_RS21440 |
| RS21440- BamHI-R | TAATGCAGATGCACGCGTCCCAG |  |
| RS21440-BamHI-F | TAATGGATCCTCGTCACAGCGTATTGCACCAC |  |
| RS21440-PstI-R | TAATCTGCAGGTTTATTGTTGAGCGACAGG |  |
| RS21445-EcoRI-F | TAATGAATTCGAAGTGGGTTCTGCGCTGGTC | Construction of pUC18_ΔECA_RS21445 |
| RS21445- BamHI-R | TAATGGATCCCGAGGCCAACCAGATGCGTTC |  |
| RS21445-BamHI-F | TAATGGATCCGCCATCACTCAGCTCGATCAGG |  |
| RS21445-PstI-R | TAATCTGCAGCTGGTACCTGTCATGCACTTCGC |  |
| RS21455-BamHI-F | taatggatCCACACAAGCAGTGATAACTGGG | Construction of pUC18_ΔECA_RS21455 |
| RS21455-PstI-R | taatctgcagCTTTCTAGCTGAACGCGACCC |  |
| RS21455-PstI-F | taatctgcagGCAGGTGACTACACTCTCCAG |  |
| RS21455-HindIII-R | taataagcttGTGAGTTACGCTTTACCGACG |  |
| RS21440-SphI-F | taatGCATGCGGGGTAAGAAACGAC | Construction of pUC18_PacHIG |
| RS21440-PstI-R | taatCTGCAGCATCTGAGCGAGCTTCCTGC |  |
| RS21455-PstI-F | taatctgcagGCAGGTGACTACACTCTCCAG |  |
| RS21455-HindIII-R | taataagcttGTGAGTTACGCTTTACCGACG |  |
| RS21455-NdeI-F | taatcatATGAAGAATATGAGTTTGGGAAAAATG | Construction of pBBR*pacG* and RT-PCR |
| RS21455-EcoRI-R | taatgaattcGGGTTAATCTGTCATGTGTTTAAAAG |  |
| CheA-NdeI-F | taatCATatggacatgagtgctttctatcaaac | Construction of pBBR*cheA* |
| CheA-PstI-R | taatCTGCAGCCTGTCCTACTGTTTCGCCAG |  |
| PacH-PacI-F | CAGCGCGGGAAGCAATAAC | RT-PCR analyses |
| PacH-PacI-R | CCAGATGCGTTCTACCGAAGATG |  |
| PacI-RS21450-F | CTCTCTCTCGCAAGCCCTAAAG |  |
| PacI-RS21450-R | GCGATGACCAGAGTAAAGCCAG |  |
| RS21450-PacG-F | CACACAAGCAGTGATAACTGGG |  |
| RS21450-PacG-R | GTCTTTCTAGCTGAACGCGACCC |  |
| RS21440-FW-RT-PCR | CCGTGCAGCTCATGGTCAAT |  |
| RS21440-RV-RT-PCR | CATGGCGCTTGTCGTCATCTC |  |
| RS21445-FW-RT-PCR | GCCTATGCCACGCAGGTAAC |  |
| RS21445-RV-RT-PCR | GACAACAGGCTGCTCGTGTC |  |
| ECA-gyrB-F-qPCR | GGCGTCTCTATTCGCCTGATTG |  |
| ECA-gyrB-R-qPCR | GTGGATTGGCGTCTTGTTACGG |  |
