# Supplementary material for: Chemotaxis to plant defense compounds in phytopathogens
Source: PLoS Pathog. 2026 May 20;22(5):e1014240. doi: 10.1371/journal.ppat.1014240 (PMC13215616; doi:10.1371/journal.ppat.1014240)
Supplement: S9 Table — The sequence extension containing the hexa-histidine tag is shown in bold. (DOCX) [file ppat.1014240.s027.docx]

**Table S9) Sequences of proteins used in this study.** The sequence extension containing the hexa-histidine tag is shown in bold.

| Protein name | Protein sequence |
| --- | --- |
| ECA_RS21440-LBD  (PacH-LBD) | **MGSSHHHHHHSSGLVPRGSHM**RVHLLDLSEDIESLSEKNLTSLILIQDAKSGFDAVARSVRTIGLTSDSSRIQEEKRLIDQQIALNTDILTKLYSHLSEPESRDSLDRLTQARPAYRDAVNKAVELGVSENAEERARAVQLMVNEMQITQAPVFAALDSMTELQKKRTMEMTTSAMQEARSDGNTLI |
| ECA_RS21445-LBD  (PacI-LBD) | **MGSSHHHHHHSSGLVPRGSHM**RTHLVGLGNTTDNLAKNHLANLIVLQELKDNLNVTIKATLRMLITTEKKVLEDNQKLIETTSARNAKLVTQLEENLQAKEVRNILGELQQNRTEFATVGRQSVALSLNNKQAESIELVRTQLEPIQTKLFNNLNTMIQLQKDYTTQTATNAIEESYYDGNSL |
| ECA_RS21450-LBD | **MGSSHHHHHHSSGLVPRGSHM**GRIQLDKLGENIQVLSQVRITNLLMMQEFKDNINTNAIAVRNLTMQEDDRLVQEEKTRIEEMISRNNALLSKIHDSTAEKHAQELVAELQRVRPAYSSSMANAITLAMANKNSEAQHLLLTDVRAKQDAVFNALNDMVNWQEKLTVEIANQSLKNATNAGSL |
| ECA_RS21455-LBD  (PacG-LBD) | **MGSSHHHHHHSSGLVPRGSHM**GRVQLERLGGNIQLLSQIRITNLLLMQEVKDNVNDTARAIRNMALLNDQQQMKTEKERIEKSIARNNDLLAQIRKNTVSSETKVQVATLEQALPAYINNMKKAIELAMTNQHEAFRNFLLTEVRAAQANVFTALDKMVERQKDLTVELANQSEKEALNAGT |
