# Supplementary material for: Chemotaxis to plant defense compounds in phytopathogens
Source: PLoS Pathog. 2026 May 20;22(5):e1014240. doi: 10.1371/journal.ppat.1014240 (PMC13215616; doi:10.1371/journal.ppat.1014240)
Supplement: Attachment — Submitted filename: Response to reviewer.docx [file ppat.1014240.s028.docx]

Reviewer #1:

The manuscript by Genova et al. investigates the chemotaxis response of the plant pathogen Pectobacterium atrosepticum. The authors identify important plant defense compounds as chemoreceptor ligands for three chemoreceptors and show a role of PacG in plant virulence. This is a well-written manuscript describing carefully composed and conducted experiments. The identification of specific chemoattractants and their corresponding chemoreceptors will contribute to a better understanding of plant pathogen/host interaction, which is an understudied area of research. However, the physiological relevance of this response requires additional experimental proof. It has not been shown that the plant defense compounds investigated in this study are released at sufficient concentrations to elicit a chemotaxis response of the pathogen.

Major comments:

Abstract, line 43: "Bacteria thus exploit plant defense responses by moving to compounds that are secreted at infection sites in response to pathogen attack." This statement needs to be supported by experimental evidence that the chemotaxis responses are physiologically relevant. Are these phytochemicals secreted at the infection site in sufficient concentrations to elicit a chemotaxis response in Pectobacterium atrosepticum? Otherwise, the statement needs to be omitted.

**Response: Many thanks for your time and efforts to review our manuscript, and for this favourable assessment. We see the point of this reviewer. The increase in the concentration of the plant defence compounds following pathogen attack has been reported in a number of publications. We now dedicate a section in the Discussion to this issue. These studies agree in the fact that these plant defence compounds are either undetectable or present in very low concentrations. However, in infected plants these compounds are present at levels that are well within the chemotactic response range.**

**The following section was added to the Discussion section of the manuscript.**

**“However, how do the concentrations of plant defense compounds detected in plants following pathogen attack correspond to the sensitivity of the corresponding chemotactic responses? A number of studies have quantified levels in infected and control plants. In one study cucumber plants were infected with *Pseudomonas syringae* and the concentration of salicylate and *p*-hydroxybenzoate quantified after 20 hours [99]. Whereas both compounds were either undetectable or at a concentration close to the detection threshold in uninfected control plants, salicylate and *p*-hydroxybenzoate concentrations rose to up to about 300 or 100 µM, respectively. The results of this study are very similar to an analogous study using a different model system, namely the infection of tobacco plants with the Tobaco Mosaic Virus [100]. In analogy to the above study, salicylate was undetectable in mock-inoculated leafs. However, 40 hours post-inoculation, a time before lesions become visible, the local salicylate concentration increased to up to 380 µM. The same study also showed that *P. syringae* infected tobacco plants also had largely increased salicylate levels. These concentrations are well above the onset concentrations for salicylate and *p*-hydroxybenzoate of 0.5 µM and 1 µM, respectively (Fig. 4). Another study has investigated *p*-coumaroylagmatine levels in *Arabidopsis thaliana* infected with *Alternaria brassicola* [101]. The authors show that this compound accumulated at concentrations of up to about 35 µM in infected plants, whereas its concentration in control plants was more than 10 times lower. This concentration is close to the *p*-coumaroylagmatine concentration that induced maximal chemotaxis (Fig. 4).”**

**In this section we have cited three representative articles. However, there are many other studies and, if needed, this section can be extended and further studies can be included.**

Minor comments:

Fig. 1B should be moved to supplemental materials as it is of minor importance for the conclusions of this work.

**Response: We agree. Fig. 1B has been transferred to the Supplementary Material where it is now Supp. Fig. 1.**

Fig. 5B: the shades of the two different columns are difficult to differentiate. Please choose greater grey-scale difference.

**Response: We agree. In the new version of this figure, data for feruloylagmatine are shown in a lighter grey scale.**

------------------------------------------------------------------------

Reviewer #2:

Genova et al describe a very complete and rigorous study on a cluster of four chemoreceptor genes in an important plant pathogen that are homologous to genes found in other pathogens. They present results identifying ligands for these receptors that include known plant defense compounds and demonstrate chemotaxis to some of these compounds including novel findings for two compounds (feruloylagmatine and p-coumarolylagmatine). They provide binding and structural evidence for docking and use mutants to determine that PacG plays a role in virulence. Overall, I find this to be a very well written and complete study and beyond addressing some minor points feel that it is suitable for publication.

**Response: Many thanks indeed for this very favourable assessment.**

These are what I see as the major claims in the paper--all of which I find to be supported.

1)	PacH, I, and G homologues are detected in other important plant pathogens (Table s2)

2)	PacH and PacL bind salicylate, vanillin, and p-hydroxybenzoate and PacG binds agmatine and derivative and these include known plant defense compounds (Fig.2)

3)	These can act as P. atrosepticum chemoattractants (Fig. 4)

4)	Agmatine completed with it's conjugates for PacG binding (Fig. 5)

5)	They identified the PacG binding pocket (Fig. 7)

6)	Mutation of PacG reduces P. atrosepticum virulence (Fig. 6)

Minor points:

Figure 3C. is there some growth on other compounds at 12hrs?

**Response: Yes, there is also growth on other compounds at 12 h. We have recently published a study reporting the identification of a chemoreceptor that responds specifically to phosphorylated C3 compounds (Velando et al. (2025) Genome Biology 26:260). We have conducted growth experiments using the same protocol. Of the five compounds tested, some growth was observed for glycerol 3-phosphate (Fig. 3A of this publication). These experiments were conducted to assess the capacity of the compounds to serve as sole carbon source. In the same publication we have also assessed the capacity of these compounds to serve as sole phosphorus source. As shown in Fig. 3B of this publication, significant growth was observed at 12 hours for 3-phosphoglycerate, glycerol 3-phosphate and glycerol 2-phosphate. It is likely that many other compounds would result in growth after 12 hours. However, we have not assessed this issue in detail**

Line 166 I do not see capric acid shown in Figure 2B

**Response: We see the point of this reviewer. In Fig. 2B we show only the titration curves of the compounds that bound. We have now added Supp. Fig 5 that shows the titration raw data with capric acid. The observed heat changes are very small and uniform indicative of an absence of binding. The thermal shift assay produces at times false-positive hits as we have discussed in our recent review (Monteagudo-Cascales et al. (2025) FEMS Microbiol Rev. 49:fuaf033). Because of unknown reasons, capric acid is a compound that has resulted in the past frequently in false-positive hits in the analysis of other proteins.**

Figure S4 would be nice for the authors to annotate the diagnostic chemical shifts vs. the reference.

**Response: We agree. A new version of this figure is now included.**

Line 195 given that the protein appears to bind agmatine in the conjugates the discussion starting on line 425 seems a bit obvious.

**Response: We agree. The sentence has been changed from “Binding of a ligand to a receptor usually triggers a response, but we show here an example of a ligand acting as an antagonist.” to “We report here a ligand that acts as an antagonist.”**

Line 465 seems like it should be 'is caused by decreased chemotaxis OR slower growth'

**Response: Thanks for spotting this. This was a typo.**

Line 466 seems like this could be a separate paragraph

**Response: We agree. A new paragraph now starts with “There is increasing evidence ….”**

Line 648 seems like these methods are incomplete

**Response: We agree. The last sentence now reads “Aliquots (10 μL) were spotted onto protease assay plates (LB broth containing 1 % (w/v) skimmed milk) that were incubated at 28 ºC during 72 h, and then inspected visually.”**

I note that this paper has an unusually large number of references. I don't have an issue with this but there may be some opportunity to be a bit more selective.

**Response: We agree. The number of references has been reduced.**
